# Effect of Different PTFE Coatings Applied to 18CrNiMo7-6 Steel on the Coefficient of Friction and Wear Under Dry Sliding Contact Using the Ball-on-Disk Method at Different Loads

**DOI:** 10.3390/polym18161991

**Published:** 2026-08-15

**Authors:** Michal Krbata, Marcel Kohutiar, Mariana Janeková, Branislav Hoferica, Daniel Krizan, Jana Escherova, Andrej Dubec, Bohdan Trembach, Pavol Mikuš, Alena Breznicka

**Affiliations:** 1 Faculty of Special Technology, Alexander Dubcek University of Trenčín, 911 06 Trenčín, Slovakia; jana.escherova@tnuni.sk (J.E.); pavol.mikus@tnuni.sk (P.M.); alena.breznicka@tnuni.sk (A.B.); 2 Faculty of Industrial Technologies in Púchov, Alexander Dubček University of Trenčín, Ivana Krasku 491/30, 020 01 Púchov, Slovakia; mariana.janekova@tnuni.sk (M.J.); branislav.hoferica@student.tnuni.sk (B.H.); andrej.dubec@tnuni.sk (A.D.); 3 Business Unit Coil. Voestalpine Steel Division GmbH, Research and Development Department, Voestalpine-Strasse 3, A-4020 Linz, Austria; daniel.krizan@voestalpine.com; 4 Chief Designer Department of Mining and Press-and-Forging Equipment, Private Joint Stock Company “Novokramatorsky Mashinostroitelny Zavod”, 04070 Kyiv, Ukraine; btrembach89@gmail.com

**Keywords:** PTFE, coating, tribology, coefficient of friction, wear

## Abstract

This study investigates the tribological performance of three commercial PTFE-based Xylan^®^ coatings—Xylan^®^ 1425, Xylan^®^ 1052, and Xylan^®^ 1010—applied to 18CrNiMo7-6 steel under dry sliding conditions. Ball-on-Disk tests were conducted at normal loads of 5, 7.5, and 10 N, wear-track radii of 12, 16, and 20 mm, and corresponding sliding velocities of 0.31–0.52 m·s^−1^. The tribological evaluation was complemented by measurements of coating thickness, surface roughness, nanoindentation, wear-track profilometry, scanning electron microscopy, EDS mapping, and post-test cross-sectional microscopy. All coatings reduced the coefficient of friction from approximately 0.49–0.64 for the uncoated steel to 0.09–0.13, corresponding to an average reduction of 78–80%. Xylan^®^ 1425 exhibited the highest nanohardness of 57.02 MPa, the highest reduced elastic modulus of 3.33 GPa, and the most favorable elastoplastic indices. It also achieved the lowest wear, with a volumetric loss of approximately 0.03 mm^3^ under the most severe conditions, representing a reduction of more than 99% compared with the substrate. Xylan^®^ 1010 provided the lowest friction but showed pronounced plastic deformation, whereas Xylan^®^ 1052 exhibited fragmentation and increased wear. Post-test cross-sectional microscopy confirmed local exposure of the steel substrate in both coatings. Overall, Xylan^®^ 1425 provided the best balance of low friction, mechanical stability, coating continuity, and wear resistance.

## 1. Introduction

Reducing friction and wear in moving machine components is one of the key prerequisites for improving their operational reliability, energy efficiency, and service life. In applications where the use of liquid lubricants is either impractical or undesirable, polymer coatings containing solid lubricants represent an important alternative. Among these materials, polytetrafluoroethylene (PTFE) occupies a particularly important position because of its low surface energy, chemical inertness, thermal stability, and very low interlamellar shear strength. These properties reduce the adhesive component of friction and promote the formation of a low-shear transfer layer on the counterbody surface [1].

Despite its low coefficient of friction, unfilled PTFE exhibits relatively low hardness, pronounced viscoelastic–plastic deformation, and a high wear rate under concentrated contact loading. Blanchet and Kennedy reported that the wear mechanism of PTFE is associated mainly with subsurface deformation, crack initiation, and the subsequent detachment of lamellar material fragments. Suitable fillers can suppress this process by interrupting the propagation of subsurface damage and stabilizing the deformed layer [2]. For this reason, PTFE is frequently used in engineering applications as a constituent of composite coatings containing a polymer binder, solid lubricants, and reinforcing or functional particles. The resulting tribological response of PTFE coatings is governed not only by the fluoropolymer content, but also by the nature of the binder system, degree of cross-linking, coating thickness, surface roughness, and distribution of solid particles. In a nanotribological study of industrial PTFE coatings, Podestà et al. demonstrated that additives and environmental conditions significantly affect both the coefficient of friction and adhesion forces at the micro- and nanoscale [3]. Demas and Polycarpou likewise showed that the lifetime of PTFE coatings intended for compressor applications depends on the combined effects of coating mechanical integrity, contact geometry, and the ability to form a stable tribofilm [4]. A transfer layer formed on the metallic counterbody plays a key role in the dry sliding behavior of PTFE. Ye et al. distinguished running-in, transition, and steady-state stages, and associated the achievement of an ultralow wear rate with the formation of a thin, continuous, and strongly adherent transfer film [5]. In a subsequent review, the same authors emphasized that the mere presence of a transfer layer does not guarantee low wear; its morphology, continuity, adhesion to the counterbody, and resistance to secondary removal are decisive [6]. Li et al. found that the thickness and stability of PTFE transfer films depend on normal load, sliding speed, and counterface morphology [7]. The importance of load and speed was also confirmed by Xie et al., who observed an increase in transfer-layer thickness in Ni/PTFE composites with increasing sliding speed and normal force. Once a stabilized regime was reached, the transfer-layer thickness changed only slightly, while directly influencing both the coefficient of friction and volumetric wear [8]. Lv et al. showed that, in nanoparticle-modified PTFE composites, changes in load can alter not only the severity of mechanical damage but also the progress of tribochemical reactions and the structure of the resulting tribofilm [9]. Consequently, a linear relationship between increasing normal load and wear cannot be assumed, particularly for coatings capable of plastic compaction and transfer-layer reorganization.

A substantial improvement in tribological performance can be achieved by introducing solid lubricants such as molybdenum disulfide, MoS_2_. Its lamellar crystal structure enables easy interlayer sliding, thereby reducing shear resistance within the contact. However, in PTFE/MoS_2_ systems, the resulting properties depend on the spatial distribution of both phases, their interaction with the polymer matrix, and the resistance of the binder to fragmentation. Amenta et al. confirmed that reinforced PTFE materials can provide a favorable combination of low friction and enhanced wear resistance, with the stability of the film transferred to the steel counterbody playing a decisive role [10]. Saisnith and Fridrici demonstrated that the mechanical and tribological properties of PTFE coatings are also sensitive to temperature and the surrounding environment. Increasing temperature modifies the compliance of the polymer layer, its ability to accommodate plastic deformation, and the stability of the transfer film [11]. Wang et al., in dry sliding tests of a PTFE coating on cemented carbide, recorded a marked reduction in friction and adhesive damage, but also pointed to the possible development of abrasive wear, delamination, and spalling once coating continuity was disrupted [12]. Similarly, Vasconcelos et al. observed that the presence of PTFE in Ni-P-PTFE composite coatings led to smoother wear-track morphologies and reduced crack formation and loose wear debris [13]. Fiołek et al. showed that increasing the content of a low-shear component in PTFE-containing polymer composite coatings can significantly reduce the coefficient of friction; however, the final durability of the coating depends on its adhesion, homogeneity, and mechanical stability [14]. Ball-on-Disk and Pin-on-Disk methods are therefore suitable tools for the comprehensive evaluation of such coatings, as they allow the evolution of friction force to be monitored while also enabling subsequent analysis of volumetric wear, wear-track topography, and damage mechanisms. Nair et al. emphasized that the correct interpretation of these tests requires consideration of load, track radius, sliding speed, counterbody material, and contact geometry [15,16,17,18].

Industrial Xylan^®^ coatings are composite systems in which fluoropolymer and solid-lubricant phases are dispersed within a polymer binder. However, direct comparisons of different commercial Xylan^®^ grades applied to the same substrate and evaluated under identical tribological conditions remain insufficiently documented. Xylan^®^ 1425 and Xylan^®^ 1052 were selected because both contain combined PTFE/MoS_2_ solid-lubricant systems but differ in their recommended operating ranges and expected mechanical performance. Xylan^®^ 1010 was included as a PTFE-based commercial reference without the same combined PTFE/MoS_2_ system. From an application perspective, these coatings are relevant to plain-bearing and bushing surfaces, valve stems and springs, sealing rings, pump components, and threaded connections operating under high contact pressures, stick–slip conditions, corrosive environments, or limited access to liquid lubricants. Such components are commonly used in energy, petrochemical, water-treatment, automotive, and general mechanical-engineering applications [19]. Their comparison under identical conditions enabled the relationships between coating architecture, nanomechanical response, solid-lubricant distribution, friction, and wear to be evaluated independently of the substrate and testing configuration. Accordingly, this study compares the coating thickness, surface roughness, nanomechanical properties, coefficient of friction, volumetric wear, damage morphology, and elemental redistribution of the three coatings applied to 18CrNiMo7-6 steel.

## 2. Materials and Methods

The base material used in this study consisted of 18CrNi bearing steel samples provided by thyssenkrupp rothe erde Slovakia, a. s., located in Považská Bystrica (Slovakia). The chemical composition of the experimental steel samples was verified using a Q4 TASMAN spectral analyzer (Bruker BioSpin GmbH, Ettlingen, Germany) and a SPECTROMAXx LMX10 device (SPECTRO Analytical Instruments GmbH, Kleve, Germany), and the obtained results are presented in Table 1.

**Figure 1:**
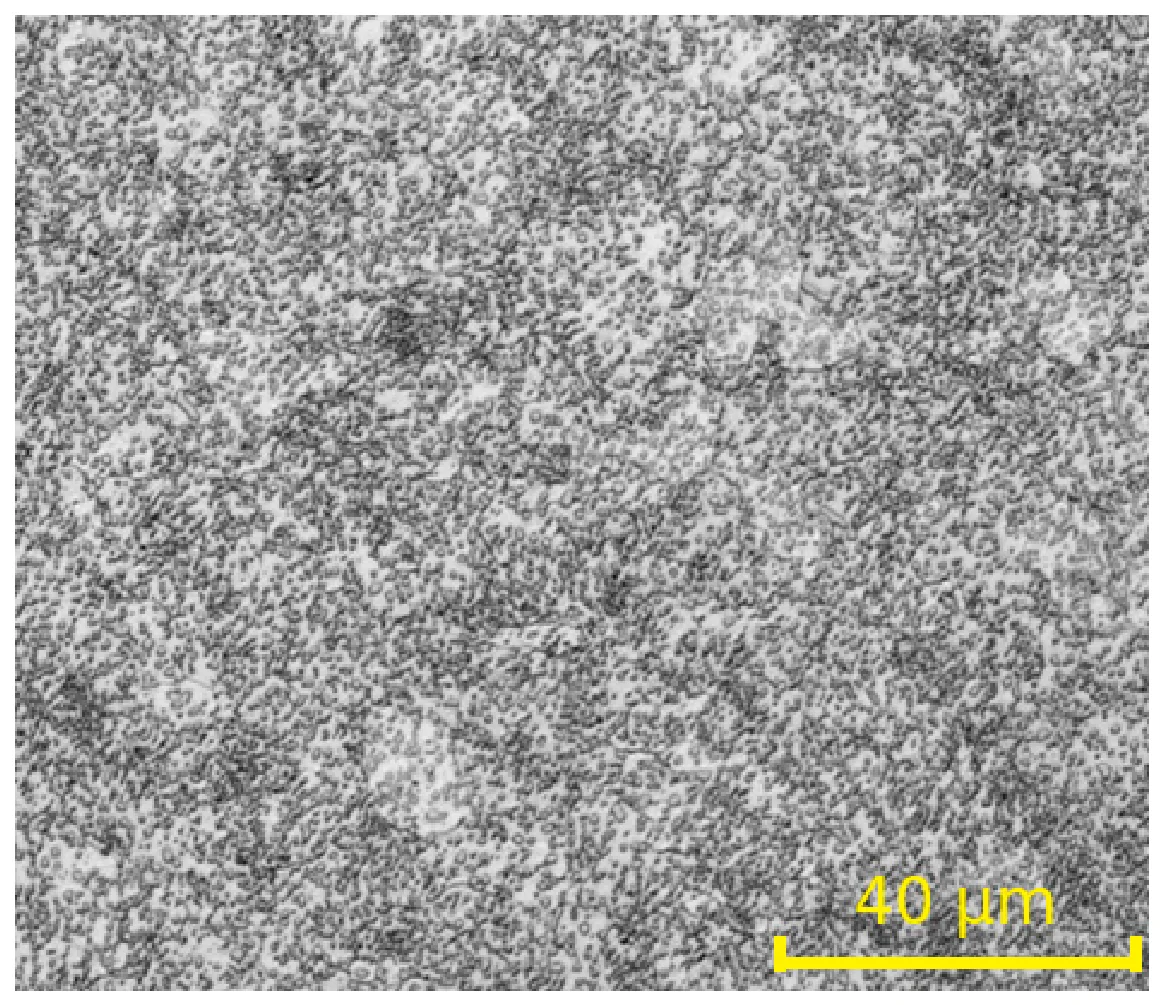
Microstructure of the base material 18CrNi.

Based on metallographic observations, the microstructure of the 18CrNi base material after etching with 3% Nital was identified as a ferritic–pearlitic structure with partially spheroidized carbides (Figure 1). The presence of pearlite and globular carbides indicates a normalized annealing.

**Figure 2:**
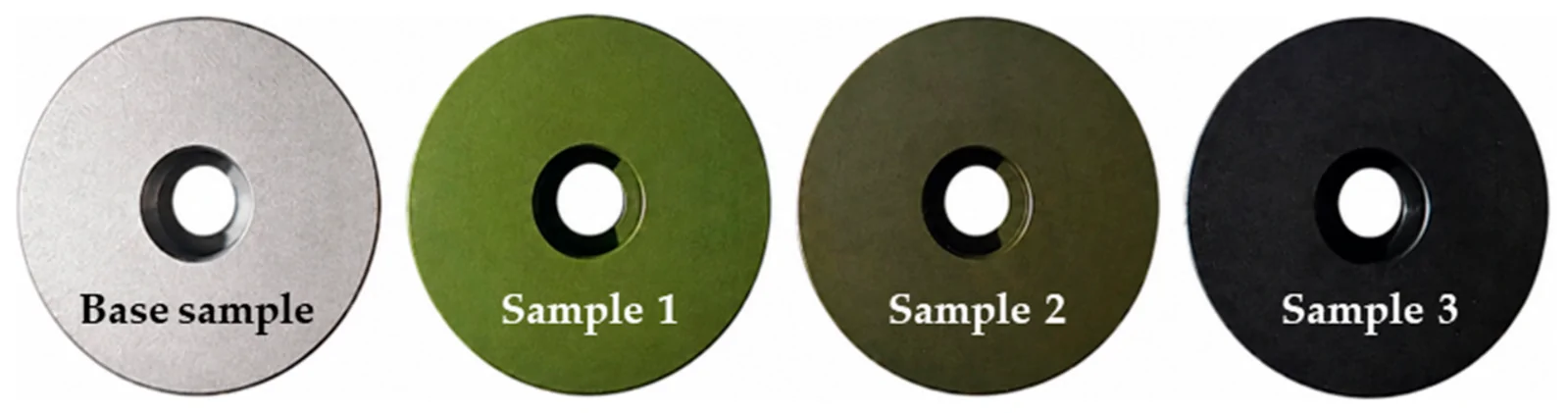
Macroscopic comparison of the base sample and modified samples after surface treatment.

Subsequently, polymer coatings based on PTFE, commercially known as Xylan^®^, were applied to the prepared samples. The designation of the individual experimental samples and the applied PTFE/Xylan coatings is presented in Table 2. The coating process was carried out by Technicoat s.r.o. in the Czech Republic using powder coating technology on a pre-treated surface. Polytetrafluoroethylene (PTFE) is a fluoropolymer with the chemical formula [CF_2_–CF_2_]_n_. It is characterized by low surface energy, high chemical stability, a low coefficient of friction, and self-lubricating properties, making it suitable for tribologically stressed functional surfaces. Within the experiment, three types of coatings were used: Xylan^®^ 1010, Xylan^®^ 1052, and Xylan^®^ 1425. The Xylan^®^ 1052 and Xylan^®^ 1425 coatings contain a MoS_2_/PTFE bonding system, which contributes to improved self-lubricating properties and wear resistance. The detailed chemical composition, binder formulation, filler content, and specific processing parameters of the commercial Xylan^®^ coatings constitute proprietary intellectual property and trade secrets of the manufacturer. Consequently, more detailed information regarding their formulation and manufacturing process could not be disclosed or obtained. Nevertheless, the available technical information and EDS results enabled the principal differences between the coatings to be identified. Xylan^®^ 1425 and Xylan^®^ 1052 are resin-bonded dry-film lubricant systems containing both PTFE and MoS_2_, whereas Xylan^®^ 1010 is primarily PTFE-based and no Mo was detected in its EDS maps. Because the exact binder chemistry, constituent fractions, additive concentrations, and curing-related cross-linking remain proprietary, the coatings were evaluated as complete commercial systems rather than as compositionally controlled formulations. Consequently, the observed differences cannot be attributed to a specific constituent concentration, and the mechanistic interpretation is based on the experimentally measured surface, nanomechanical, elemental-distribution, and tribological characteristics. Therefore, the coatings are identified by their commercial designations and were characterized experimentally in terms of thickness, surface roughness, nanomechanical properties, elemental distribution, and tribological performance. A macroscopic comparison of the base sample and the modified samples after surface treatment is shown in Figure 2.

### 2.1. Tribological Test

The tribological behavior of the prepared specimens was evaluated using the Ball-on-Disk method on a Bruker UMT TriboLab tribometer (Bruker Corporation, Billerica, MA, USA). The experiments were conducted under dry sliding conditions, i.e., without the application of external lubrication, in order to isolate and accurately assess the intrinsic influence of the PTFE/Xylan coatings on the frictional response and wear resistance of the investigated surfaces. During testing, the counterbody performed a continuous circular sliding motion over the specimen surface under a defined normal load and constant rotational speed, thereby generating a controlled tribological contact under steady-state conditions. The evolution of the coefficient of friction (COF) was continuously monitored throughout the test, while the resulting wear tracks were subsequently subjected to detailed post-experimental characterization focused on wear morphology, damage mechanisms, and surface degradation behavior.

A precision steel ball with a diameter of 4.76 mm and a G40 accuracy grade was used as the counterbody, reflecting the anticipated operating conditions in which PTFE-based coatings are predominantly exposed to contact with metallic counterparts. The G40 designation represents the dimensional and geometrical precision grade of the ball rather than its chemical composition. Balls of this class are commonly manufactured from high-carbon chromium bearing steel AISI 52100 (equivalent to 100Cr6/1.3505), characterized by high hardness, excellent rolling contact fatigue resistance, and good dimensional stability [20,21]. The chemical composition of the ball material is presented in Table 3. The hardness of the steel counterbody ball reached approximately 805 HV1.

The operating conditions and parameters of the performed tribological tests, including the applied normal load, wear track radius, sliding velocity, rotational speed, and test duration, are summarized in Table 4.

The normal loads of 5, 7.5, and 10 N were selected to provide three progressively increasing levels of contact severity while avoiding immediate catastrophic penetration of the 20–40 µm coating layers. For reference, a Hertzian calculation for the uncoated steel–steel contact, using a 4.76 mm ball and E = 210 GPa and ν = 0.30 for both bodies, gives maximum contact pressures of approximately 1.32, 1.51, and 1.66 GPa, respectively. These values represent reference upper estimates because the greater compliance of the polymer coatings increases the actual contact area. The track radii of 12, 16, and 20 mm produced sliding velocities of 0.31, 0.42, and 0.52 m·s^−1^ and total sliding distances of approximately 188, 251, and 314 m during the 600 s tests. This parameter range enabled the effects of increasing normal load and sliding velocity to be evaluated under low-to-moderate dry-sliding conditions relevant to plain-bearing surfaces, bushings, sealing elements, valves, and other components operating without continuous liquid lubrication.

To enable a direct comparison between the different loads and wear-track radii, the specific wear rate was calculated according to k = V/(F_N_L), where V is the measured volumetric loss, F_N_ is the applied normal load, and L is the total sliding distance. The sliding distance was calculated as L = 2πrnt, where r is the wear-track radius, n is the rotational speed, and t is the test duration. The resulting sliding distances were approximately 188, 251, and 314 m for track radii of 12, 16, and 20 mm, respectively. The specific wear rates are expressed in mm^3^·N^−1^·m^−1^.

### 2.2. Measurement of Layer Thickness and Roughness

The thickness of the deposited PTFE/Xylan coatings and the surface topography characteristics were analyzed using a 3D confocal laser scanning microscope, Olympus LEXT OLS5100 (EVIDENT Europe GmbH, Hamburg, Germany), enabling non-contact and non-destructive evaluation of surface morphology with high spatial resolution [22]. The coating thickness was determined on prepared cross-sections of the coated specimens, from which metallographic mounts were produced. The thickness of the individual coating layers was subsequently evaluated by microscopic analysis of the cross-sectional area in order to verify compliance with the manufacturer-declared coating thickness range of 20–40 μm. The resulting coating thickness was determined as the arithmetic mean of repeated measurements performed at different locations along the analyzed cross-section.

The experimental characterization also included the evaluation of surface roughness and topographical changes before and after tribological loading. The analysis was focused on comparing the morphology of the original surface with the wear track generated during the Ball-on-Disk tribological testing using a steel counterbody ball. The acquired 3D topographical data were processed using the dedicated software (version 3.1.1.8472) of the measuring system, enabling detailed analysis of surface geometry evolution and degradation mechanisms of the coating layers after tribological loading.

To determine the residual coating thickness after tribological testing and to verify possible local exposure of the steel substrate, selected specimens tested at a normal load of 10 N and a wear-track radius of 20 mm were sectioned perpendicular to the wear track. The cross-sectional specimens were mounted in metallographic resin, polished, and examined using the same Olympus LEXT OLS5100 confocal microscope (Tokyo, Japan). The residual coating thickness was measured at three representative positions in each cross-section. This analysis was also used to identify local coating discontinuities and to clarify the origin of the Fe-rich metallic regions observed within the wear tracks.

### 2.3. Nanohardness

The nanomechanical properties of the base material and the applied PTFE/Xylan coatings were evaluated using the quasistatic nanoindentation method in order to analyze the local mechanical response of the surface layers. The measurements were performed using a Hysitron TriboIndenter TI 950 system [22,23]. Prior to indentation, the morphology of the analyzed regions was recorded using the integrated Scanning Probe Microscopy (SPM) mode, enabling precise localization of individual indents with nanometer-scale lateral resolution. Nanoindentation tests were carried out within areas of 10 × 10 μm using a standard trapezoidal loading function with a maximum load of 500 μN and an indentation time of 2 s. A diamond Berkovich indenter TI-0039 with a three-sided pyramidal geometry, an included angle of 142.3°, and a tip radius of approximately 100 nm was used for the measurements, providing detailed evaluation of hardness and elastic response of thin coating systems. The evaluated output parameters were nanohardness H [GPa] and reduced Young’s modulus Er [GPa].

### 2.4. SEM

The microstructural and chemical characterization of the specimens with the applied PTFE/Xylan coatings was performed using scanning electron microscopy (SEM) combined with energy-dispersive X-ray spectroscopy (EDS). The analysis was carried out using a JEOL 7600 FEG microscope (JEOL Ltd., Tokyo, Japan) equipped with an Oxford Instruments INCA microanalysis system and an X-Max 50 EDS detector (Oxford Instruments, Abingdon, UK) [24]. The measurements were conducted at an accelerating voltage of 15 kV. The SEM observations focused primarily on evaluating the surface morphology, coating-layer continuity, and the characteristics of the wear tracks. The EDS analysis was aimed mainly at elements associated with the composition of the steel substrate and the PTFE/Xylan coatings, particularly C, O, F, Fe, Mo, and S. This approach enabled the spatial distribution of the individual elements to be examined and allowed the coating homogeneity, the presence of transfer layers, and local wear-induced compositional changes to be assessed. Because the EDS measurements were performed on topographically heterogeneous wear-track surfaces containing coating material, wear debris, and locally transferred steel, the elemental maps were evaluated primarily qualitatively. Reliable quantitative determination of the light elements C and F, expressed as mass or atomic percentages, was not possible from the existing mapping dataset. Furthermore, EDS cannot directly determine the chemical architecture or degree of cross-linking of the polymer binder.

## 3. Results

### 3.1. Analysis of Coating Thickness

**Figure 3:**
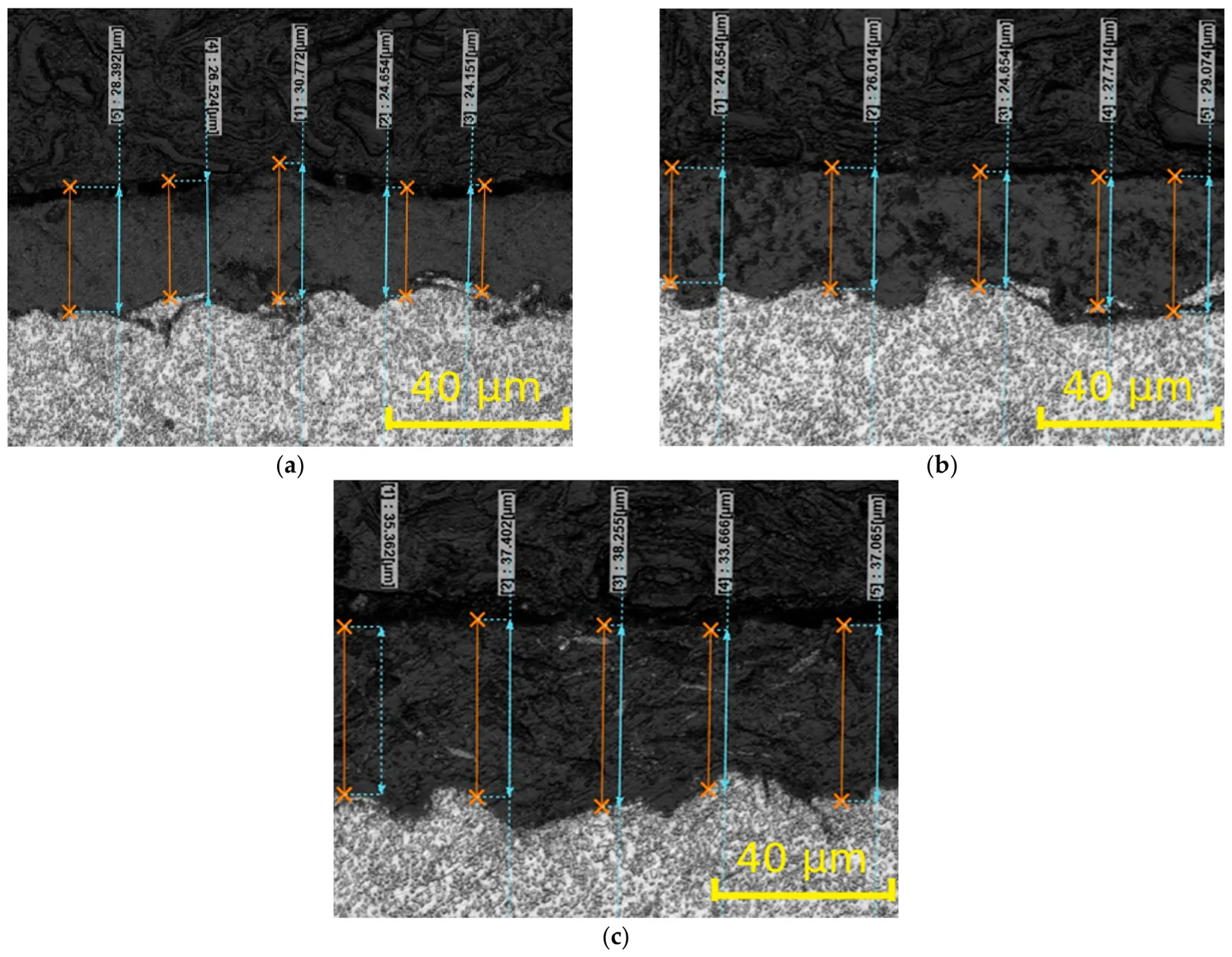
Thickness of PTFE/Xylan coatings: (**a**) Sample 1—Xylan^®^ 1425; (**b**) Sample 2—Xylan^®^ 1052; (**c**) Sample 3—Xylan^®^ 1010.

**Figure 4:**
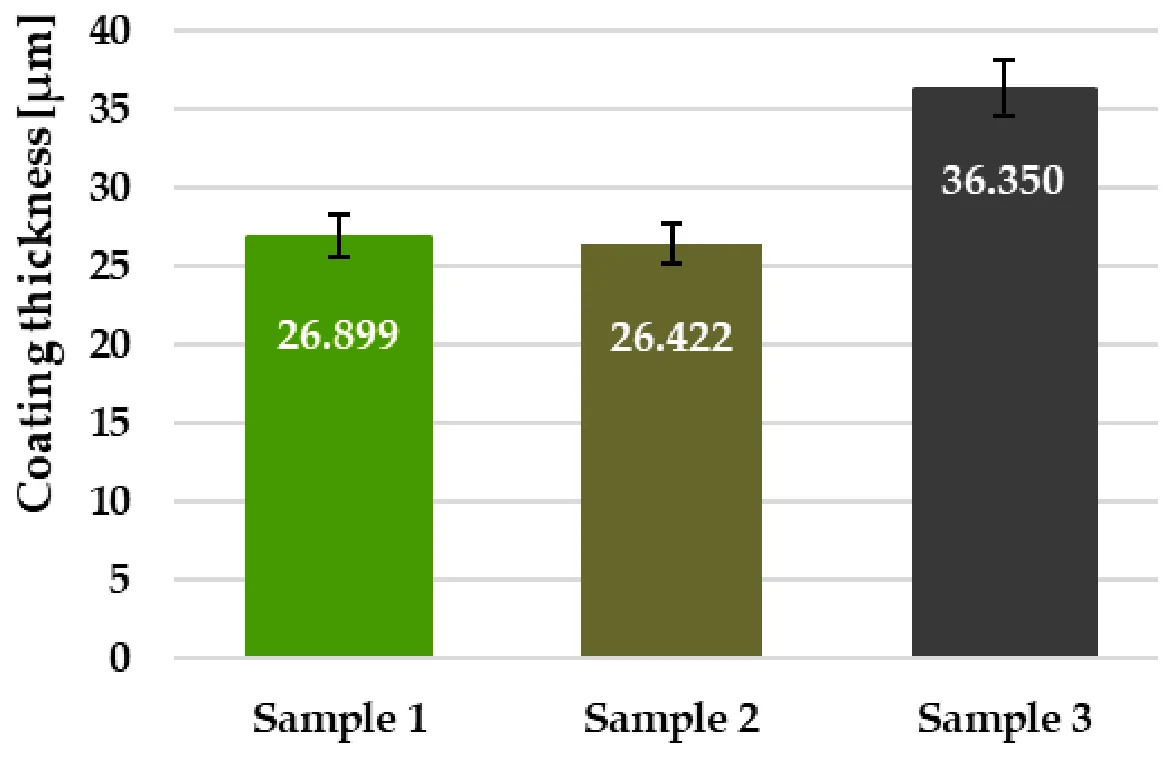
Comparison of the average thickness of PTFE/Xylan coatings.

Figure 3 shows the cross-sectional micrographs of the analyzed PTFE/Xylan coatings together with the indicated coating thickness measurements. The average thickness values of the individual coatings are summarized in Figure 4. The average thickness of the PTFE/Xylan coatings reached 26.899 ± 1.345 μm for Sample 1 coated with Xylan^®^ 1425, 26.422 ± 1.321 μm for Sample 2 coated with Xylan^®^ 1052, and 36.350 ± 1.812 μm for Sample 3 coated with Xylan^®^ 1010. The Xylan^®^ 1425 and Xylan^®^ 1052 coatings exhibited comparable coating thicknesses, with only a minimal difference between them. In contrast, the Xylan^®^ 1010 coating achieved a significantly greater layer thickness, approximately 9.5–9.9 μm higher compared to the other analyzed coatings.

All evaluated PTFE/Xylan coatings formed continuous and homogeneous layers within the technologically required thickness range. Based on the morphology of the cross-sectional areas, it was also observed that the applied coatings partially eliminated the original surface irregularities of the steel substrate and created a more compact and uniform surface morphology. The coating layers did not fully replicate the topography of the base material but locally filled surface depressions and asperities, which may positively influence the character of the tribological contact. The increased thickness of the Xylan^®^ 1010 coating may contribute to improved separation of the tribological contact between the steel substrate and the steel counterbody during the Ball-on-Disk test, potentially leading to a reduction in adhesive interaction and wear intensity. However, this assumption must be further verified based on the results of subsequent tribological and microscopic analyses.

### 3.2. Roughness and Nanohardness

**Figure 5:**
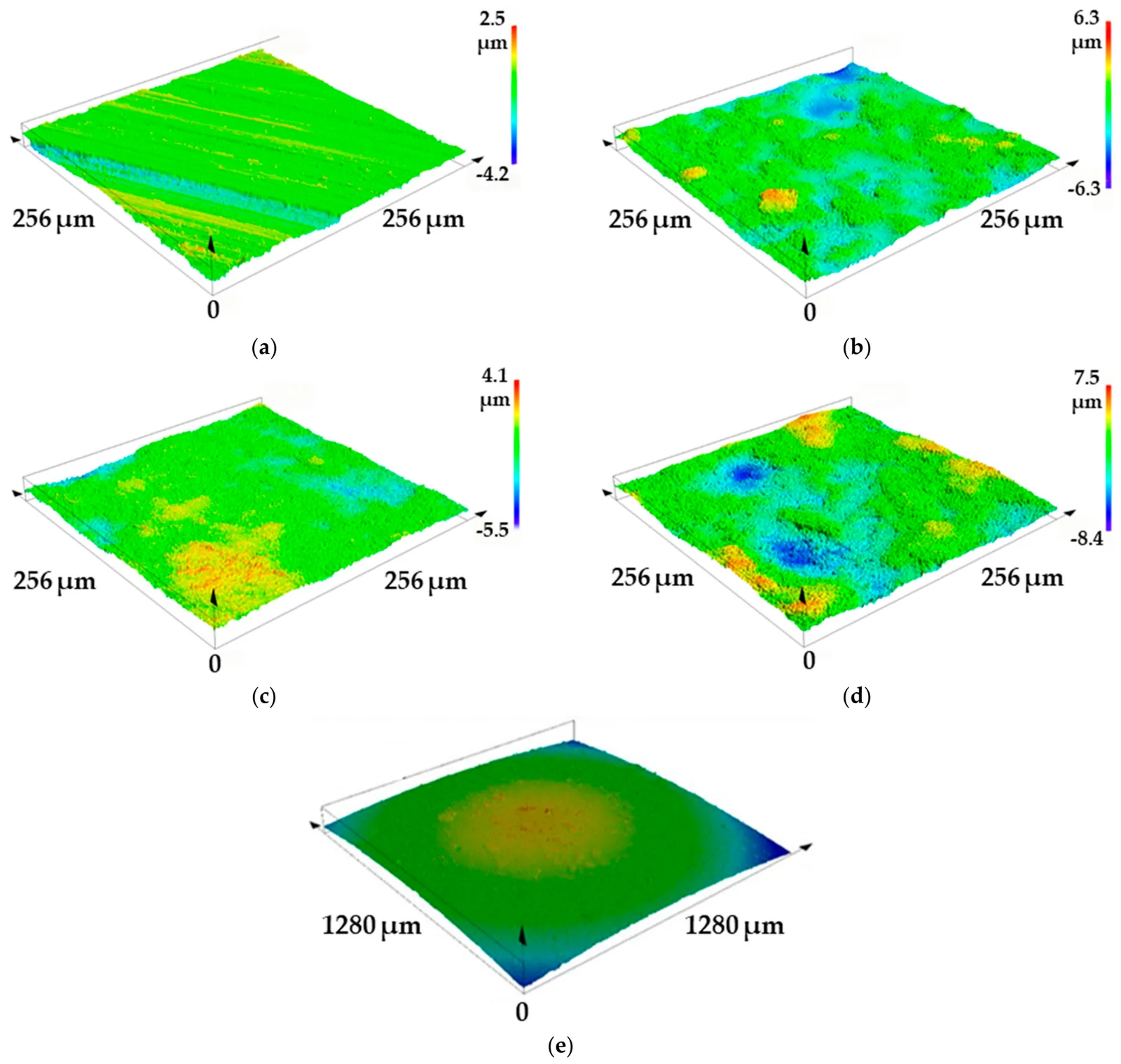
Surface texture assessed using a confocal microscope: (**a**) Base Sample − Sa = 0.41 µm; (**b**) Sample 1 −U Xylan^®^ 1425 − Sa = 0.98 µm; (**c**) Sample 2 − Xylan^®^ 1052 − Sa = 0.63 µm; (**d**) Sample 3 − Xylan^®^ 1010 − Sa = 1.57 µm; (**e**) G40 steel − Sa = 0.32 µm.

**Figure 6:**
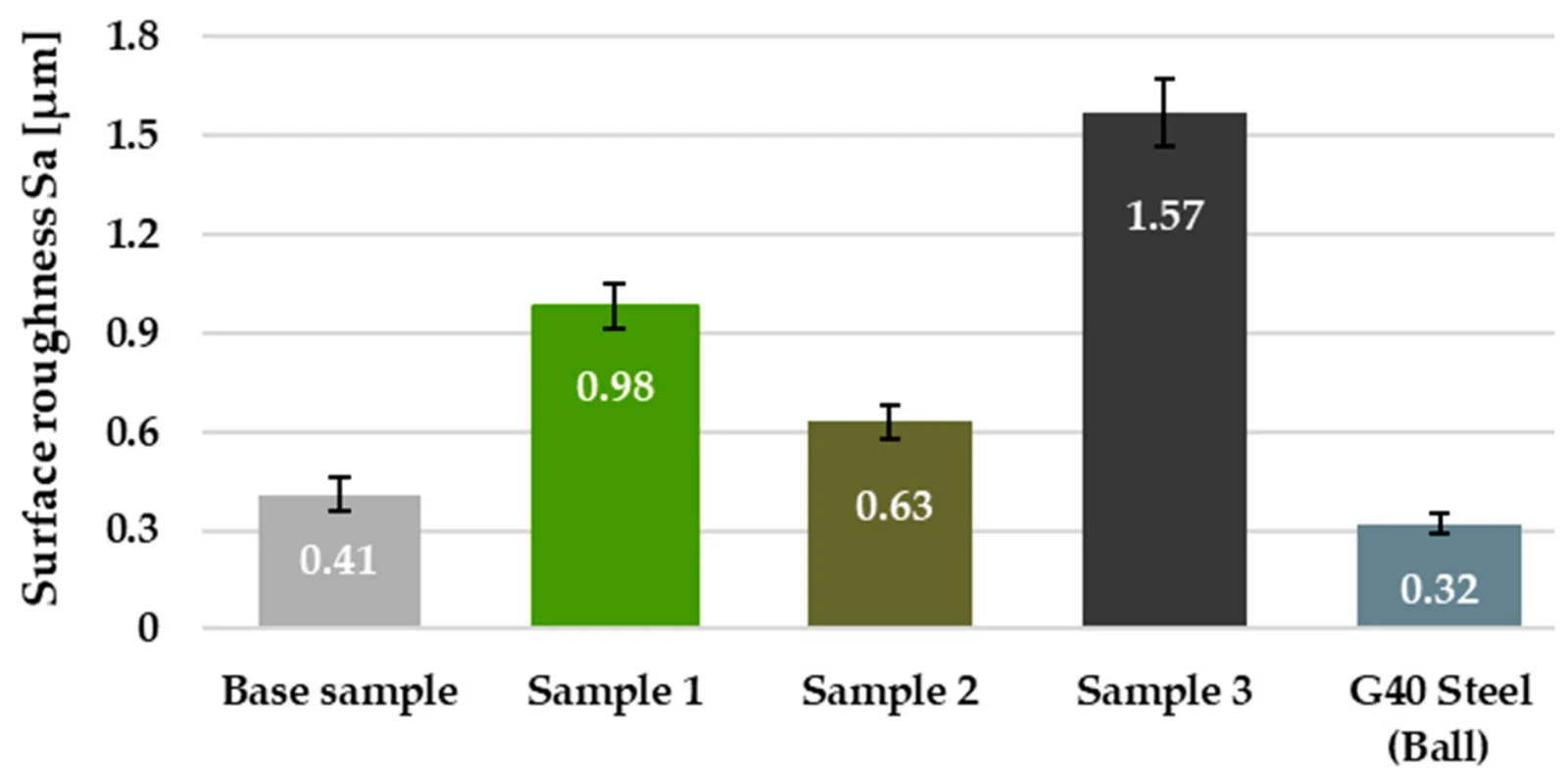
Comparison of the average surface roughness of the analyzed PTFE/Xylan coatings, base material, and G40 steel counterbody ball.

Figure 5 shows representative areas of surface roughness measurements obtained by confocal microscopy for the base material, the individual PTFE/Xylan-coated samples, and the surface of the steel counterbody ball. The evaluation was performed using the arithmetical mean height parameter Sa, which characterizes the average amplitude of surface irregularities within the analyzed area. The overall comparison of the measured surface roughness values for all analyzed surfaces is presented in Figure 6.

The base material exhibited a relatively low surface roughness value of Sa = 0.41 µm (Figure 5a), corresponding to a homogeneous surface after prior mechanical preparation performed by surface grinding. The surface of the base material was characterized by uniformly oriented grinding marks without the presence of significant local defects or pronounced surface asperities. Sample 1 coated with Xylan^®^ 1425 reached a surface roughness value of Sa = 0.98 µm (Figure 5b). Compared to the base material, a more pronounced surface texturing and a higher amplitude of surface irregularities were observed, which can be associated with the deposition and curing characteristics of the polymer PTFE coating. For Sample 2 coated with Xylan^®^ 1052, a surface roughness value of Sa = 0.63 µm was measured (Figure 5c). The surface exhibited a more compact and homogeneous morphology with a lower degree of surface irregularities compared to the other analyzed coatings, indicating a more favorable topographical character of the formed coating layer. The highest surface roughness was recorded for Sample 3 coated with Xylan^®^ 1010, where the Sa parameter reached 1.57 µm (Figure 5d). The increased surface roughness may be related to the greater coating thickness, enhanced surface texturing, and the local formation of more pronounced surface asperities during the coating deposition and curing process. Considering the significantly higher roughness of this coating system, it can be assumed that Sample 3 may also exhibit the highest wear intensity during tribological loading due to the increased contribution of asperity interactions within the contact zone. However, this assumption must be verified based on the results of subsequent tribological and microscopic analyses.

The surface of the G40 steel counterbody ball exhibited a surface roughness value of Sa = 0.32 µm (Figure 5e), representing the lowest roughness among all analyzed surfaces. This confirms the high quality of the counterbody surface finish and minimizes the influence of the ball surface irregularities on the tribological contact conditions during the Ball-on-Disk tests.

**Figure 7:**
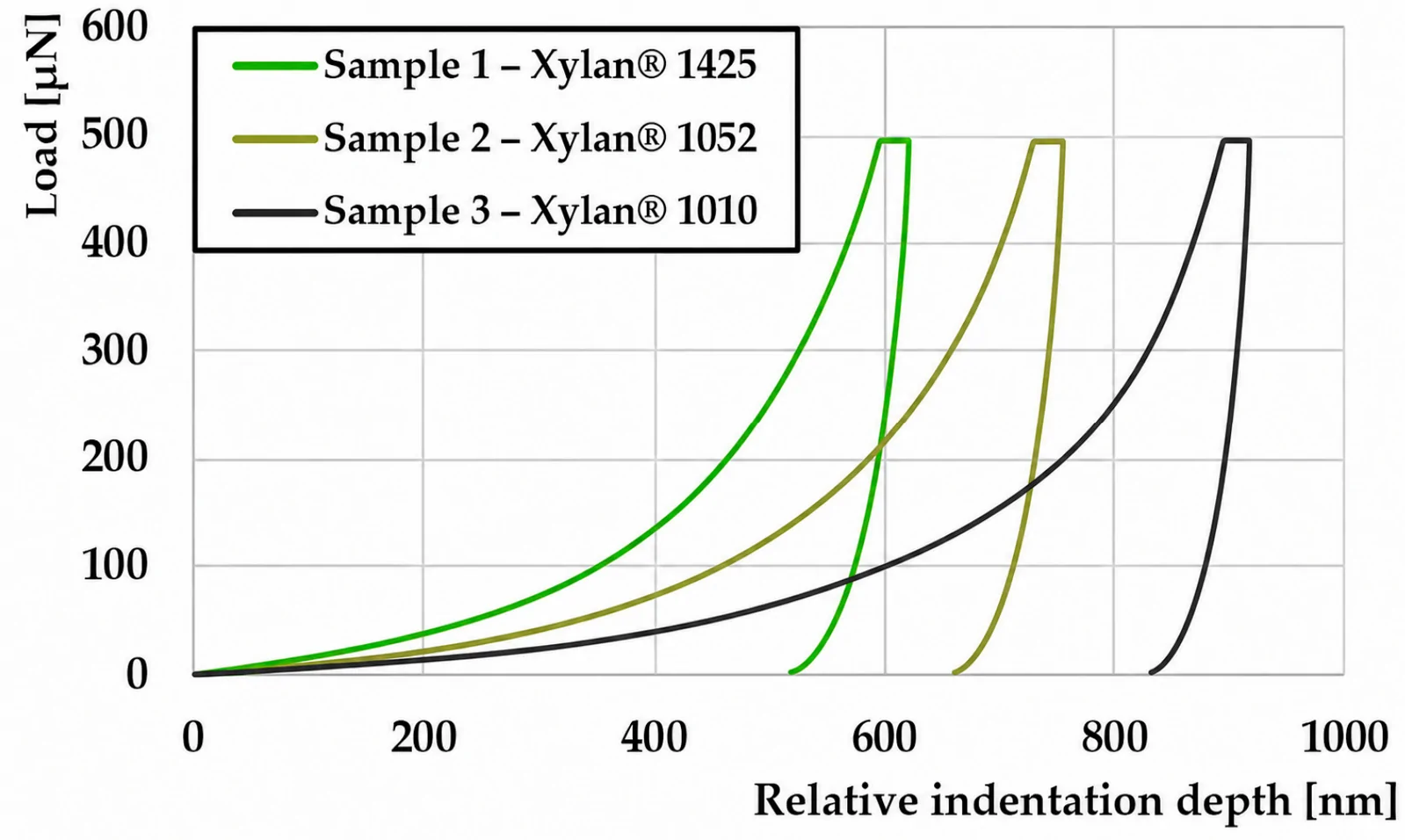
Representative nanoindentation curves of the investigated coatings.

As indicated by the data presented in Table 5 and by the representative loading–unloading curves shown in Figure 7, the individual coatings exhibited different mechanical responses under localized contact loading. The curves differed mainly in the maximum indentation depth, the slopes of the loading and unloading branches, and the magnitude of the residual deformation after unloading. The maximum indentation depth decreased in the following order: Sample 3 (Xylan^®^ 1010) > Sample 2 (Xylan^®^ 1052) > Sample 1 (Xylan^®^ 1425), which corresponds to the progressive increase in nanohardness from 25.27 MPa through 31.70 MPa to 57.02 MPa.

The greatest indentation depth and the most pronounced residual deformation were observed for Xylan^®^ 1010 (Sample 3). This behavior indicates greater compliance and a more pronounced viscoelastic–plastic character of the coating, which may be associated with the dominant contribution of the PTFE phase. PTFE exhibits low resistance to localized penetration and readily undergoes plastic flow under concentrated loading, which is reflected by the shift in the curve toward greater indentation depths and by the lower slope of the unloading branch. This interpretation is also consistent with the lowest reduced elastic modulus of 2.66 GPa and the lowest values of $H/E_{r}=0.0095$ and $H^{3}/E_{r}^{2}=2.28\times 10^{-6}$ GPa.

Xylan^®^ 1052 (Sample 2) exhibited a lower indentation depth and a slightly steeper unloading branch. Its higher hardness of 31.70 MPa and reduced elastic modulus of 2.78 GPa may be related to the presence of rigid MoS_2_ particles in the PTFE-based matrix. The lamellar MoS_2_ phase may restrict the local mobility of the polymer chains and partially increase the resistance of the coating to indenter penetration. Nevertheless, the response of Sample 2 remained markedly more compliant than that of Sample 1, indicating a lower stiffness of the binder system and a higher contribution of irreversible deformation.

The most favorable mechanical response was observed for Xylan^®^ 1425 (Sample 1). Its loading curve was shifted toward the lowest indentation depths, while the unloading branch exhibited the steepest slope, indicating the highest contact stiffness and the largest elastic contribution to the overall deformation. The nanohardness of 57.02 MPa and the reduced elastic modulus of 3.33 GPa were the highest among all the investigated coatings. This coating also exhibited the highest values of $H/E_{r}=0.0171$ and $H^{3}/E_{r}^{2}=16.72\times 10^{-6}$ GPa, confirming its superior resistance to localized and permanent plastic deformation. Since Xylan^®^ 1425, similarly to Xylan^®^ 1052, contains PTFE and MoS_2_, its substantially higher hardness and stiffness are likely related mainly to differences in the architecture of the polymer binder and to a higher degree of cross-linking after curing.

As shown by the results presented in Table 5, the calculated elastoplastic parameters $H/E_{r}$ and $H^{3}/E_{r}^{2}$ enable a more detailed assessment of the resistance of the investigated coatings to contact-induced and permanent plastic deformation. The most favorable values were obtained for the Xylan^®^ 1425 coating (Sample 1), for which H/Er = 0.0171 and $H^{3}/E_{r}^{2}=16.72\times 10^{-6}$ GPa were determined. Compared with the Xylan^®^ 1052 coating (Sample 2), the $H/E_{r}$ ratio was approximately 50% higher and the $H^{3}/E_{r}^{2}$ parameter was approximately four times greater. Relative to the Xylan^®^ 1010 coating (Sample 3), the $H/E_{r}$ ratio was approximately 80% higher, while the $H^{3}/E_{r}^{2}$ value was approximately 7.3 times greater.

These results are also consistent with the highest nanohardness measured for the Xylan^®^ 1425 coating (Sample 1), which reached 0.057 GPa (Table 5), and indicate its greater ability to sustain localized contact loading without pronounced plastic deformation. The Xylan^®^ 1052 (Sample 2) and Xylan^®^ 1010 (Sample 3) coatings exhibited lower values of both parameters, namely 0.0114 and $4.12\times 10^{-6}$ GPa, and 0.0095 and $2.28\times 10^{-6}$ GPa, respectively, indicating a greater susceptibility to localized plastic deformation. This trend was partially confirmed by the profilometric evaluation of the wear tracks, where the most pronounced deformation and material loss were observed for the Xylan^®^ 1052 coating (Sample 2).

### 3.3. Coefficient of Friction

**Figure 8:**
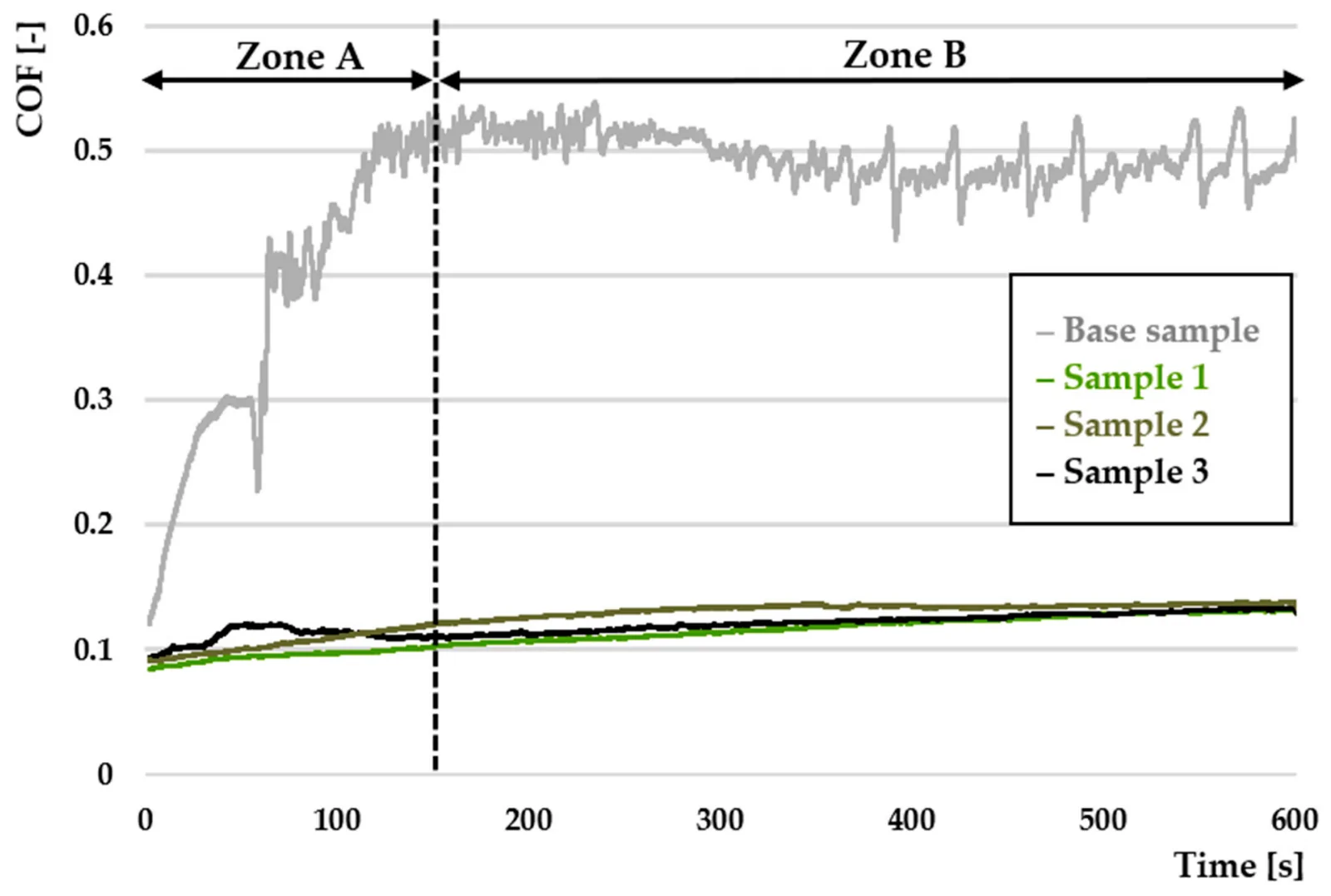
Comparison of selected friction coefficient curves.

Representative COF curves obtained under a normal load of 5 N were selected to illustrate the tribological behavior of the analyzed systems. The evolution of the coefficient of friction for the PTFE/Xylan coatings and the base material during the Ball-on-Disk test is shown in Figure 8. Based on the obtained results, the tribological response can be divided into two main regions. Region A represents the initial contact phase, characterized by a rapid increase in the coefficient of friction due to direct interaction of surface asperities, local plastic deformation, and gradual stabilization of the contact conditions. For the uncoated base material, the coefficient of friction reached values of approximately 0.5, while the friction curve exhibited more pronounced fluctuations associated with the adhesive-abrasive nature of the contact.

Region B represents the stabilized phase of the tribological contact, during which the coefficient of friction becomes more stable and a steady-state friction regime is established [25,26]. Compared to the base material, all analyzed PTFE/Xylan coatings exhibited significantly lower coefficients of friction and a more stable tribological response under the given testing conditions. This effect is mainly associated with the self-lubricating properties of the PTFE component, which contributes to the reduction in adhesive interaction and overall friction intensity during tribological loading.

**Figure 9:**
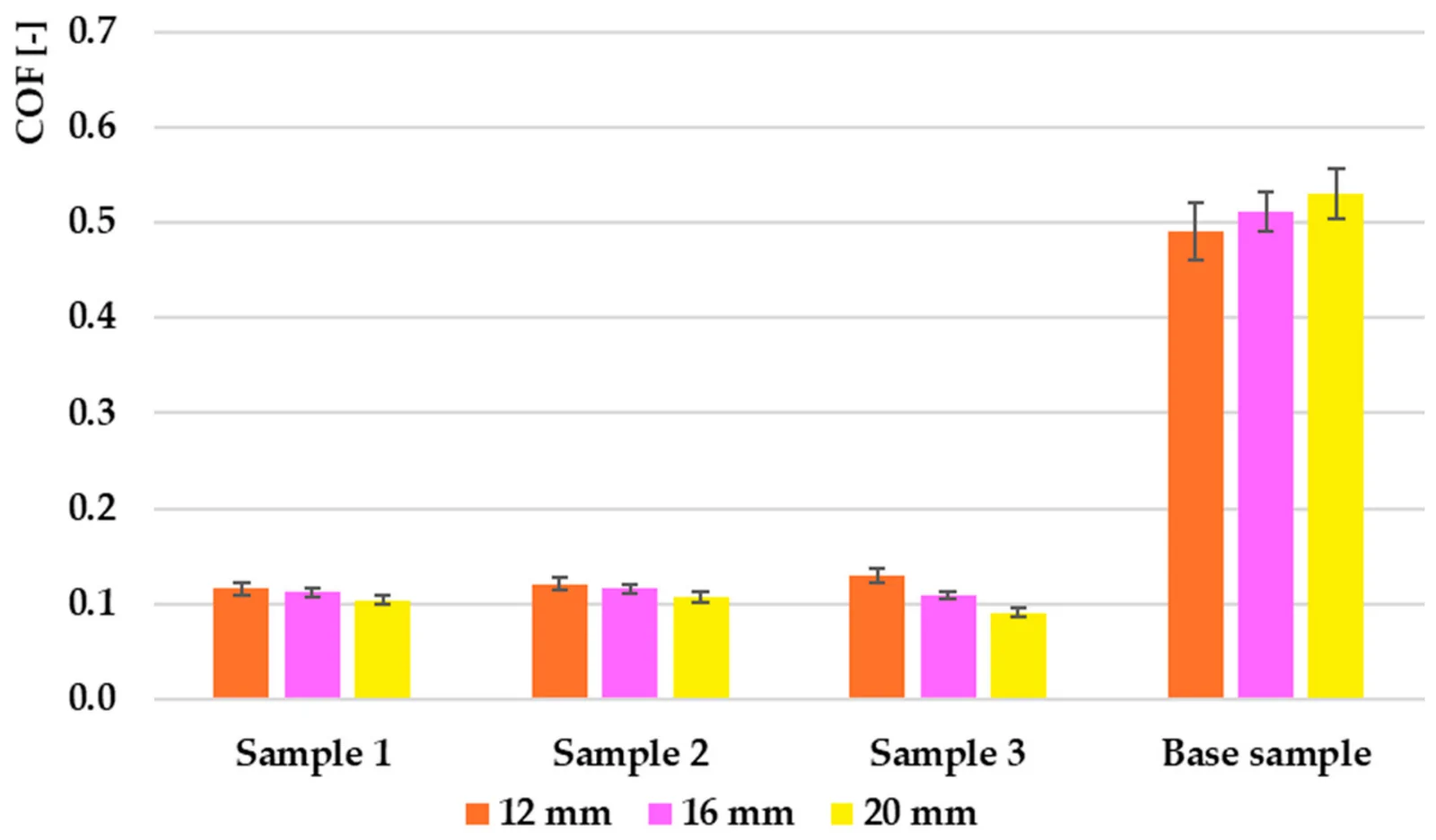
Comparison of COF at a load of 5 N.

**Figure 10:**
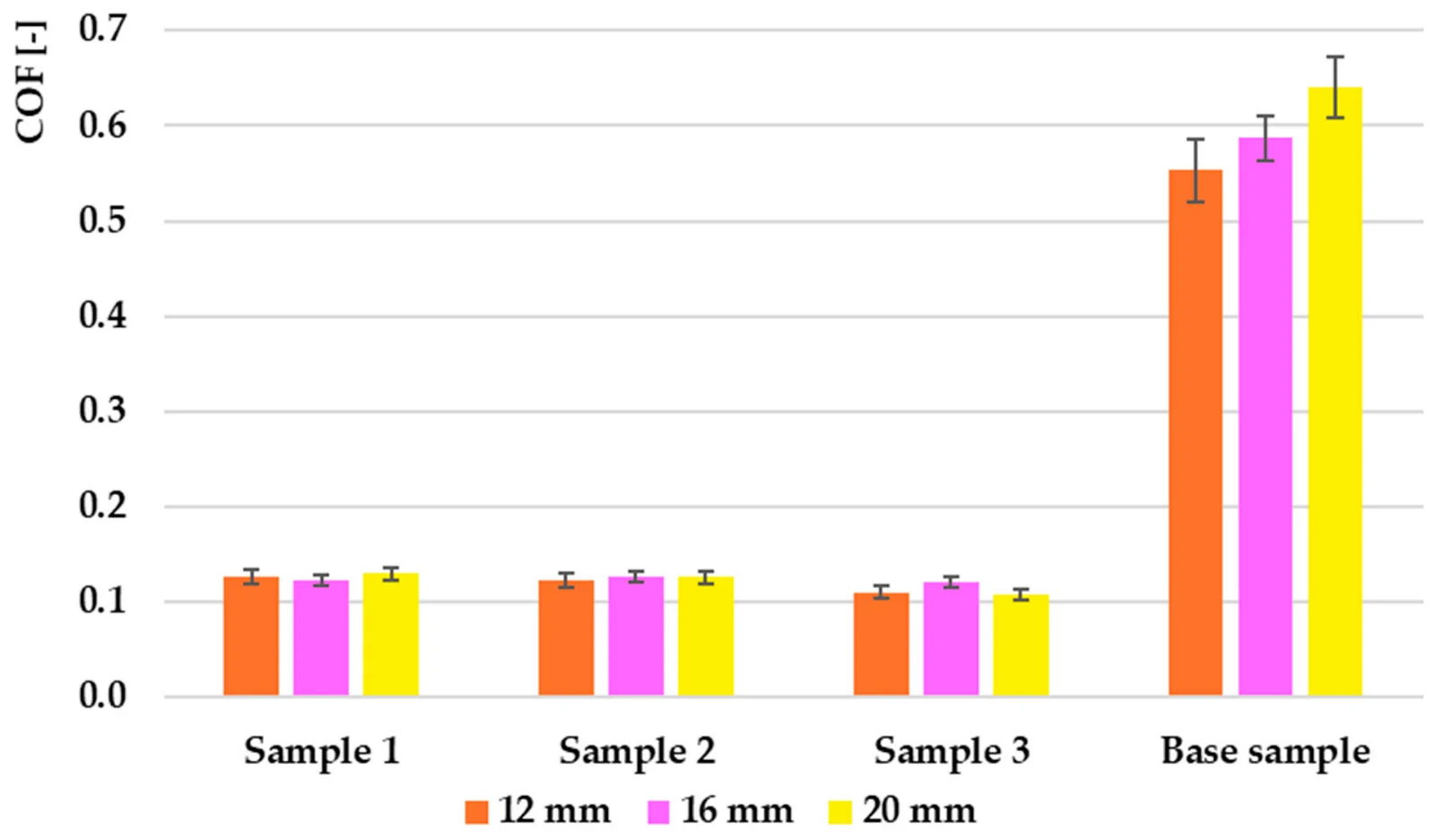
Comparison of COF at a load of 7.5 N.

**Figure 11:**
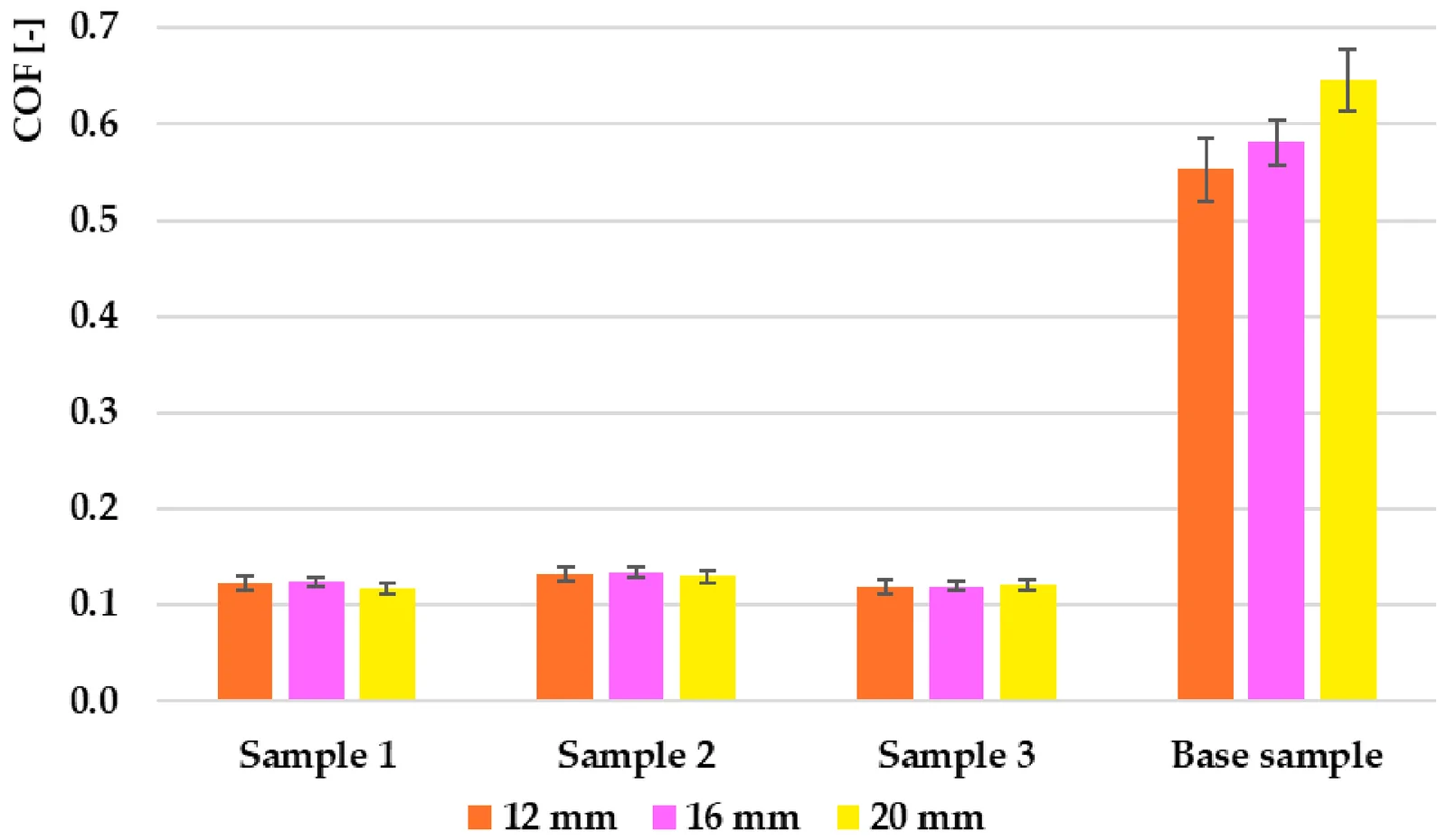
Comparison of COF at a load of 10 N.

Figure 9, Figure 10 and Figure 11 show the average values of the coefficient of friction (COF) for the analyzed PTFE/Xylan coatings and the uncoated base material under normal loads of 5 N (Figure 9), 7.5 N (Figure 10), and 10 N (Figure 11) at different wear track radii. The obtained results clearly demonstrated the significant positive effect of the PTFE/Xylan coatings on the reduction in the coefficient of friction compared to the uncoated base material. While the base material exhibited the highest COF values in the range of approximately 0.49–0.64 depending on the applied load and wear track radius, the coated samples showed substantially lower values, approximately within the range of 0.09–0.13.

For the uncoated base material, a gradual increase in the coefficient of friction was observed with increasing wear track radius and, consequently, increasing sliding velocity. This phenomenon is likely associated with a more intensive adhesive-abrasive wear mechanism, increased local thermal loading of the contact zone, and enhanced activation of adhesive interactions between the steel ball and the steel substrate. Higher sliding velocity leads to increased frictional energy generation and local temperature rise, which may destabilize oxide layers formed on the surface of the base material, intensify plastic deformation of surface asperities, and increase the real contact area. As a result of these processes, the resistance to sliding increases, leading to higher COF values.

In contrast, the PTFE/Xylan coatings exhibited the opposite trend, where increasing wear track radius generally resulted in a slight decrease in the coefficient of friction. The lowest COF values were recorded for Sample 3 coated with Xylan^®^ 1010, where the coefficient of friction reached values of approximately 0.09. This effect can be attributed to the self-lubricating behavior of the PTFE phase and the more effective formation of a stable tribofilm at higher sliding velocities. The increased contact dynamics likely promoted a more uniform distribution of the PTFE phase within the tribological contact zone, resulting in more efficient separation of the contacting surfaces, reduction in adhesive interactions, and suppression of micro-cutting effects caused by the steel counterbody ball. Simultaneously, higher sliding velocity may contribute to local orientation and compaction of the polymer layer in the sliding direction, leading to stabilization of the contact regime and a reduction in friction intensity.

The higher initial surface roughness of Xylan^®^ 1010 (Sa = 1.57 µm) may have reduced the number and size of load-bearing microcontacts during the initial sliding phase, thereby limiting adhesive interactions. However, its low nanohardness and reduced elastic modulus indicate that the surface asperities were susceptible to rapid viscoelastic–plastic deformation, flattening, and compaction during running-in. Consequently, the influence of the initial topography probably decreased as sliding progressed. The higher roughness may therefore have contributed to the low initial friction, whereas the stabilized COF was likely governed mainly by coating compliance and the formation of a PTFE-rich transfer film. Because roughness and coating formulation were not varied independently, their individual contributions cannot be quantitatively separated from the present results.

The Xylan^®^ 1425 and Xylan^®^ 1052 coatings exhibited a similar tribological response, with slightly higher COF values compared with the Xylan^®^ 1010 coating; however, their friction coefficients remained significantly lower than those of the uncoated substrate. Comparison of the results obtained under different normal loads further indicated that increasing the applied load did not have a significantly adverse effect on the tribological stability of the PTFE/Xylan coatings. Similar behavior was reported by Yuan and Yang, who observed that PTFE-based coatings maintained a low frictional response over a range of applied loads [27]. Likewise, Ren et al. demonstrated that PTFE-containing composite coatings can retain a low and relatively stable coefficient of friction under different loading conditions due to the self-lubricating action of PTFE and the formation of a stable transfer layer [28]. Even under higher contact pressures, the investigated coating systems maintained low and stable friction coefficients, indicating effective limitation of direct metal-to-metal contact and stable tribofilm formation during the Ball-on-Disk tests.

**Figure 12:**
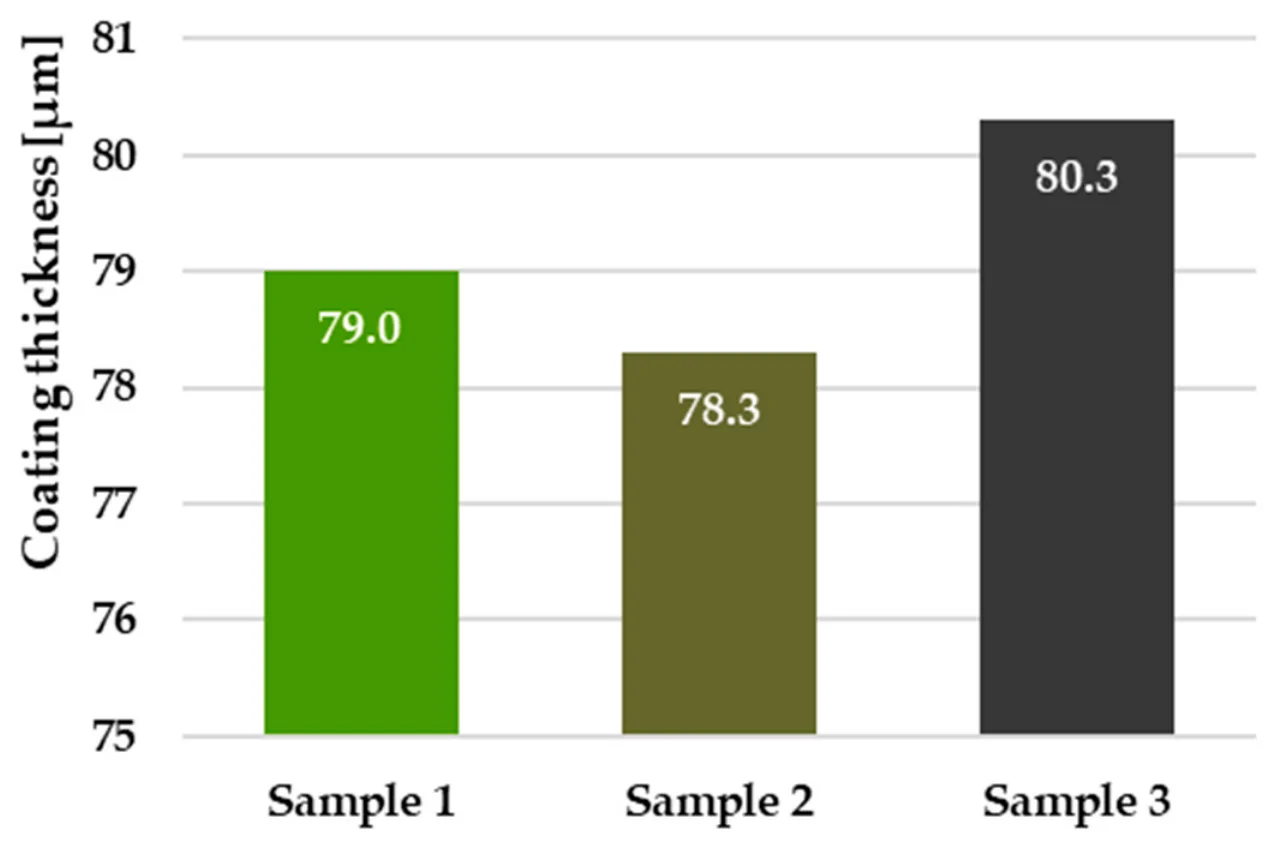
Reduction in coefficient of friction compared to uncoated base material.

Figure 12 shows the average improvement in the coefficient of friction (COF) values of the analyzed PTFE/Xylan coatings calculated from all performed tribological measurements under different normal loads and wear track radii. The presented values represent the percentage reduction in the coefficient of friction compared to the uncoated base material, which exhibited the highest COF values during the Ball-on-Disk tests. Based on the averaged results, it was found that the applied PTFE/Xylan coatings reduced the coefficient of friction by approximately 78–80% compared to the uncoated substrate. The highest average improvement in tribological performance was observed for the Xylan^®^ 1010 coating, where the COF reduction reached approximately 80.3%. The Xylan^®^ 1425 and Xylan^®^ 1052 coatings also exhibited a significant positive effect, with average reductions in the coefficient of friction of approximately 79.0% and 78.3%, respectively.

The obtained results clearly confirm the high effectiveness of the PTFE/Xylan coating systems in reducing adhesive friction and stabilizing the tribological contact under dry sliding conditions. Peng et al. similarly demonstrated that PTFE-based composite coatings can markedly reduce the coefficient of friction and wear by promoting the formation of a lubricating interfacial layer [29]. Wei et al. further showed that enhanced adhesion and cohesion of the PTFE transfer film improve its stability on the metallic counterface and contribute to sustained low-friction and low-wear behavior [30]. The significant reduction in COF values observed in the present study is therefore most likely associated with the self-lubricating properties of the PTFE phase and the formation of a stable tribofilm, which effectively separates the contacting surfaces and suppresses adhesive–abrasive wear mechanisms.

### 3.4. Wear

**Figure 13:**
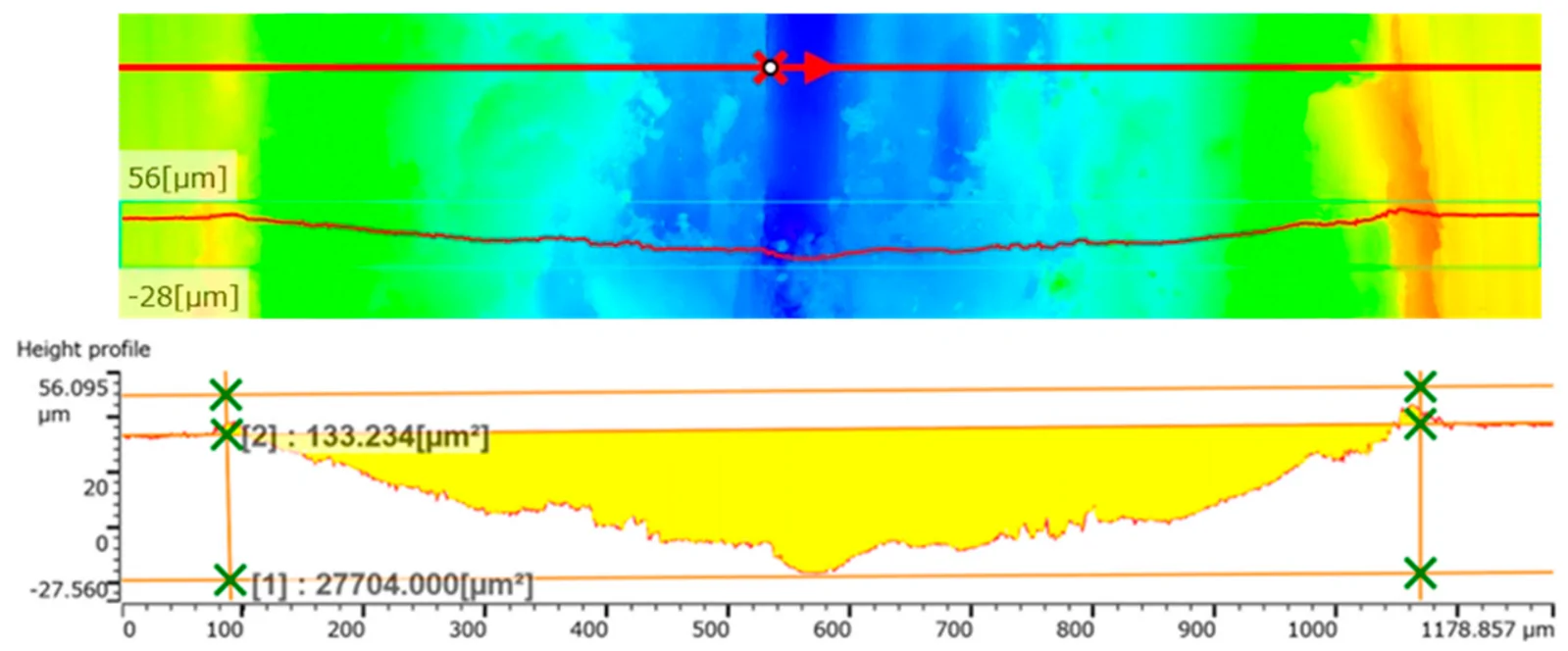
Surface topography and cross-sectional profile of a wear mark on the base material at a load of 10 N and a r = 20 mm.

**Figure 14:**
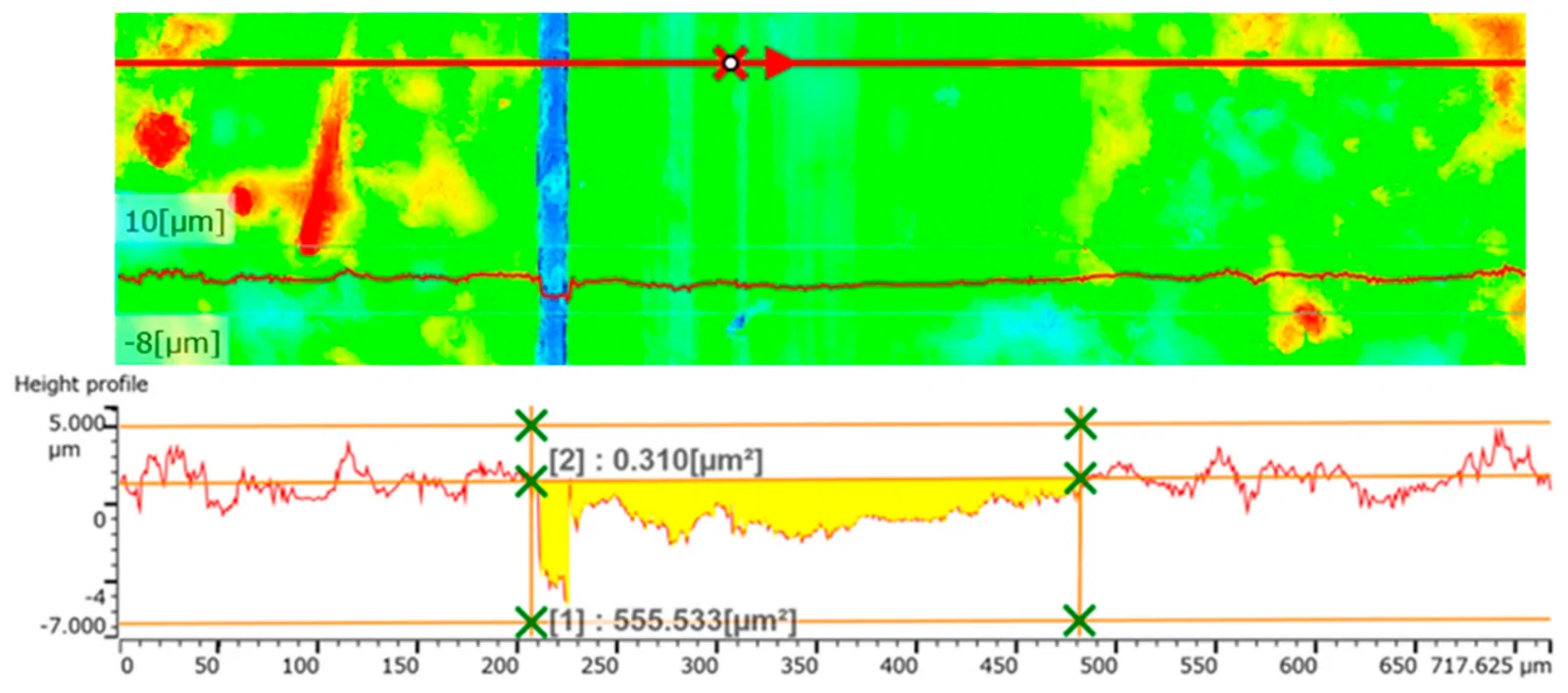
Surface topography and cross-sectional profile of the wear mark on sample 1 at a load of 10 N and r = 20 mm.

**Figure 15:**
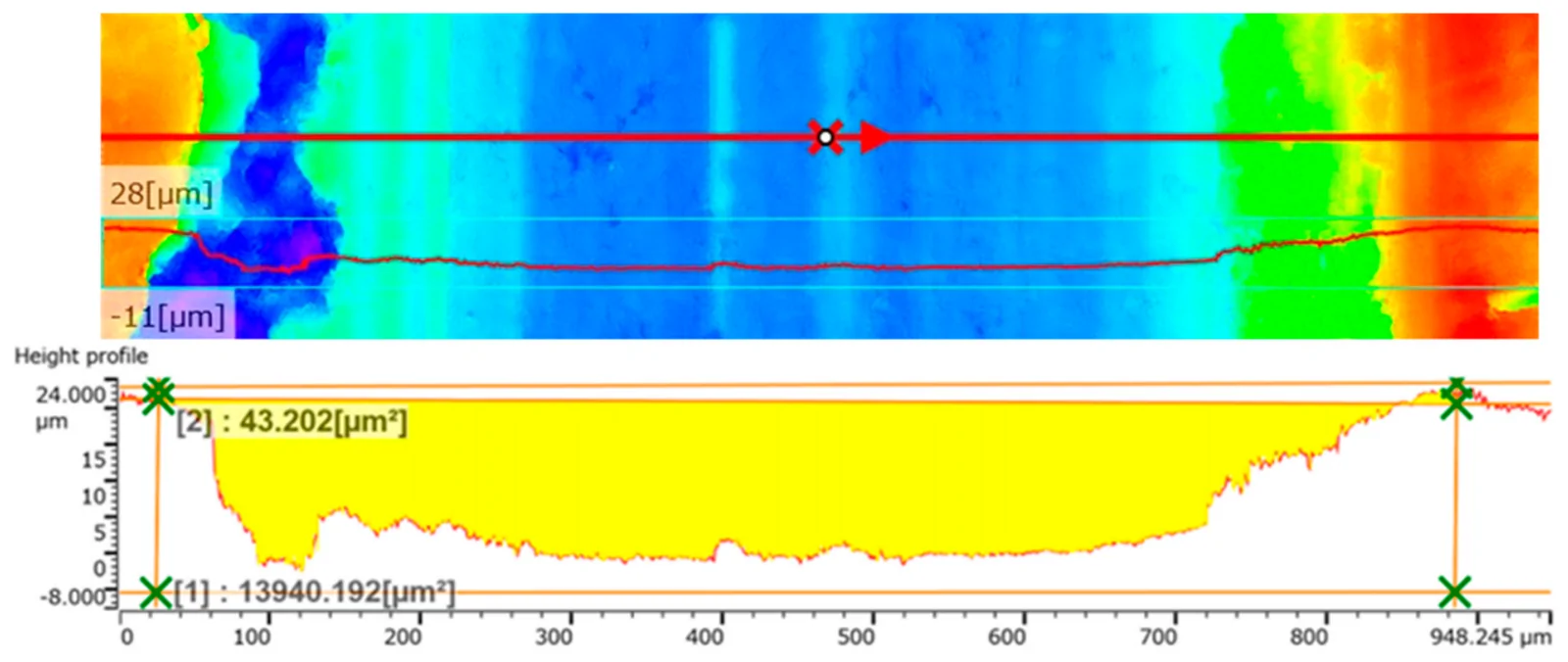
Surface topography and cross-sectional profile of the wear mark on sample 2 at a load of 10 N and r = 20 mm.

**Figure 16:**
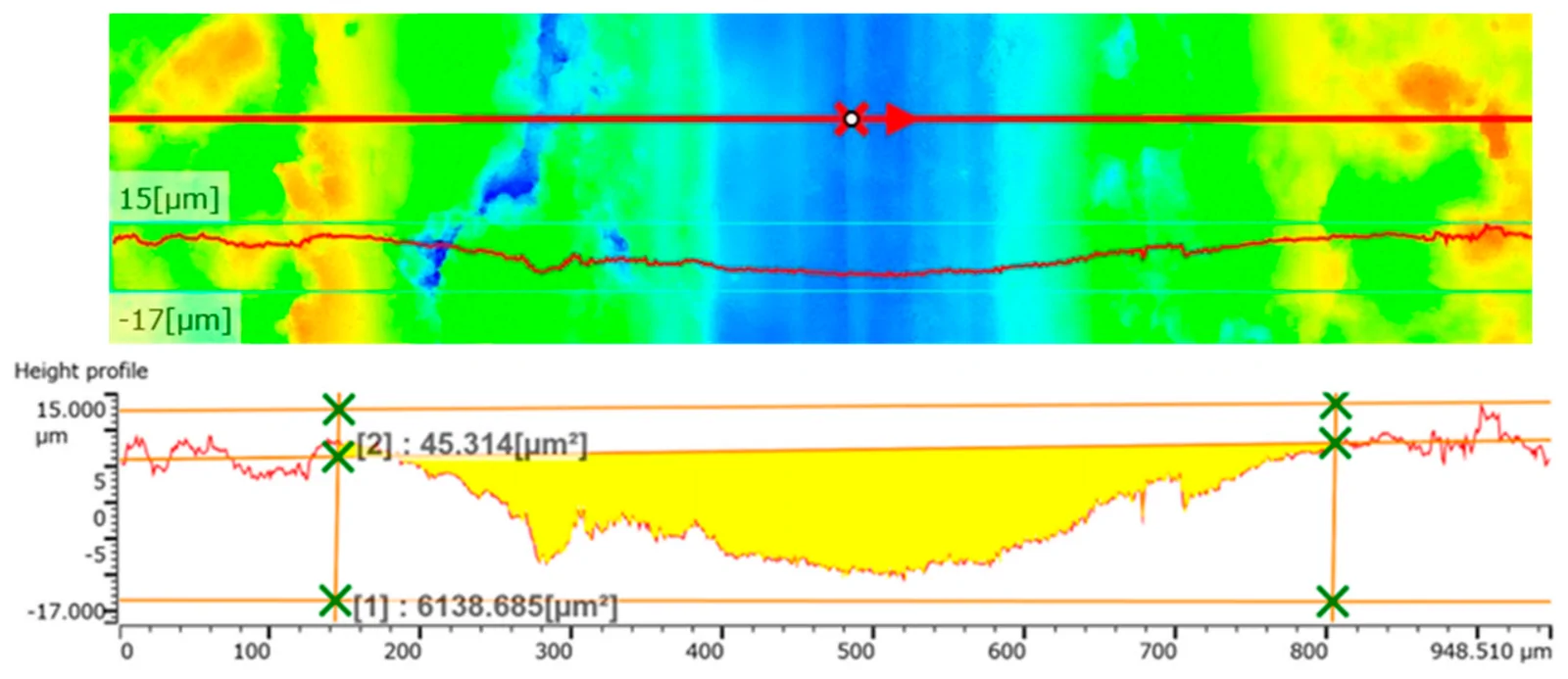
Surface topography and cross-sectional profile of the wear mark on sample 3 at a load of 10 N and r = 20 mm.

The topography and cross-sectional profiles of the wear tracks generated at a load of 10 N and a track radius of 20 mm are presented in Figure 13, Figure 14, Figure 15 and Figure 16. The uncoated substrate exhibited a wide and deep wear track with a cross-sectional area of approximately 27,704 µm^2^ (Figure 13), indicating intensive material removal accompanied by pronounced plastic plowing of the surface.

The lowest extent of damage was observed for Xylan^®^ 1425 (Sample 1). The cross-sectional area of the wear track reached only approximately 556 µm^2^ (Figure 14), representing a reduction of about 98% compared with the uncoated substrate. The wear-track profile showed smooth transitions between the unaffected surface and the worn region, without pronounced material pile-up at the track edges. Such a profile indicates limited plastic redistribution of the coating material and high structural stability under contact loading. The narrow and shallow wear track also confirms the ability of the coating to effectively separate the steel substrate from the counterbody. This result is consistent with the highest nanohardness and the most favorable $H/E_{r}$ and $H^{3}/E_{r}^{2}$ values reported in Table 5.

For Xylan^®^ 1052 (Sample 2), the cross-sectional area reached approximately 13,940 µm^2^ (Figure 15), corresponding to about 50% of the value measured for the uncoated substrate. The broad and relatively deep profile, together with distinct material pile-up on both sides of the wear track, indicates extensive plastic deformation and lateral displacement of the coating material outside the contact zone. Such edge accumulation is characteristic of pronounced plastic flow and local redistribution of the deformed surface layer.

Xylan^®^ 1010 (Sample 3) exhibited a cross-sectional area of approximately 6139 µm^2^ (Figure 16), which was about 78% lower than that of the uncoated substrate and approximately 56% lower than that of Sample 2. Pronounced pile-up was also observed along the edges of the wear track, confirming a substantial contribution of plastic deformation and material displacement during sliding. However, compared with Sample 2, the wear track was narrower and its cross-sectional area was smaller. Despite exhibiting the lowest nanohardness, Sample 3 therefore showed a more favorable profilometric response than Sample 2, indicating that the resulting wear resistance was governed not only by mechanical hardness but also by the ability of the coating to accommodate plastic deformation, redistribute displaced material, and form a stable transfer layer.

**Figure 17:**
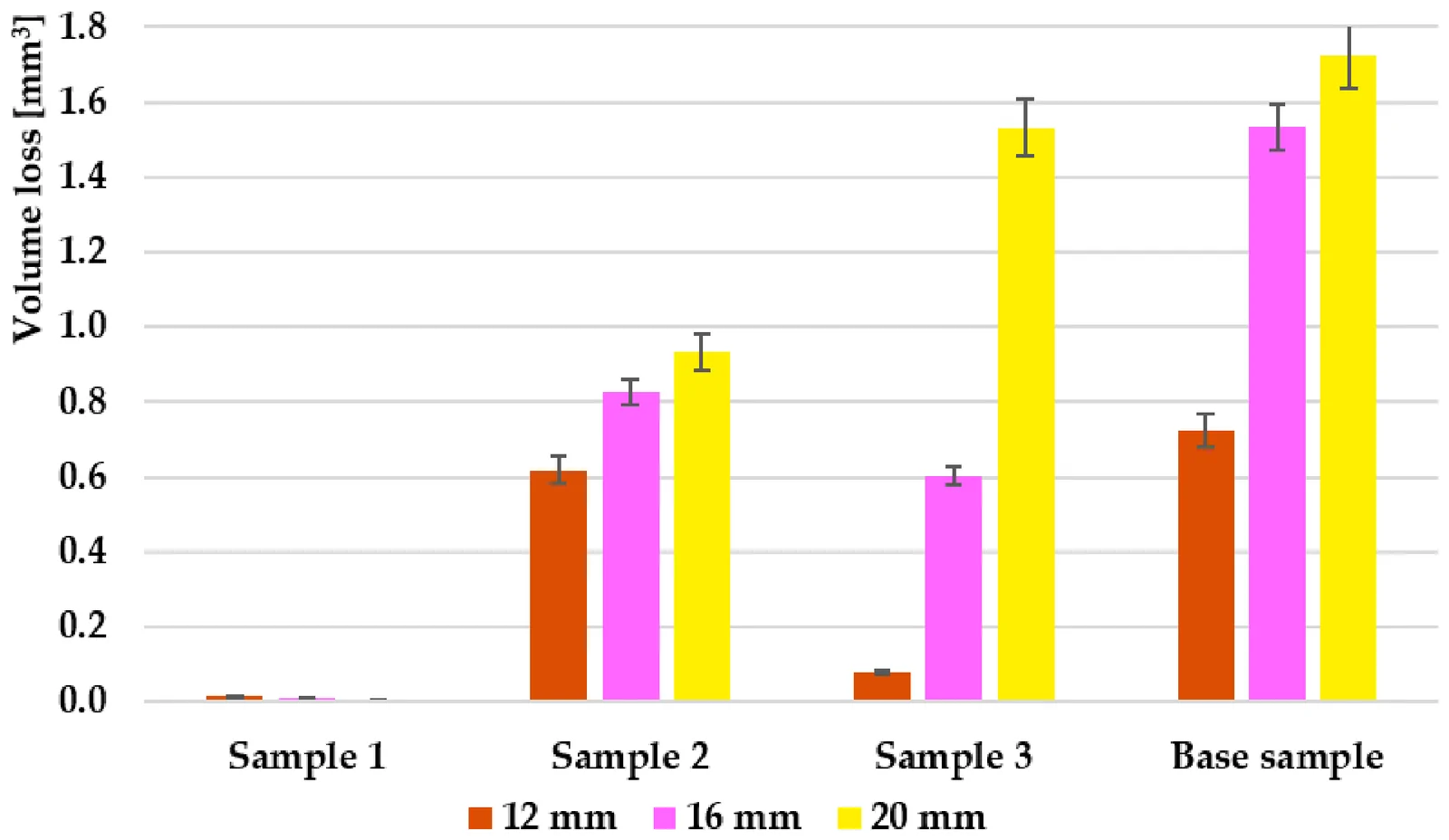
Comparison of volumetric wear at a load of 5 N.

**Figure 18:**
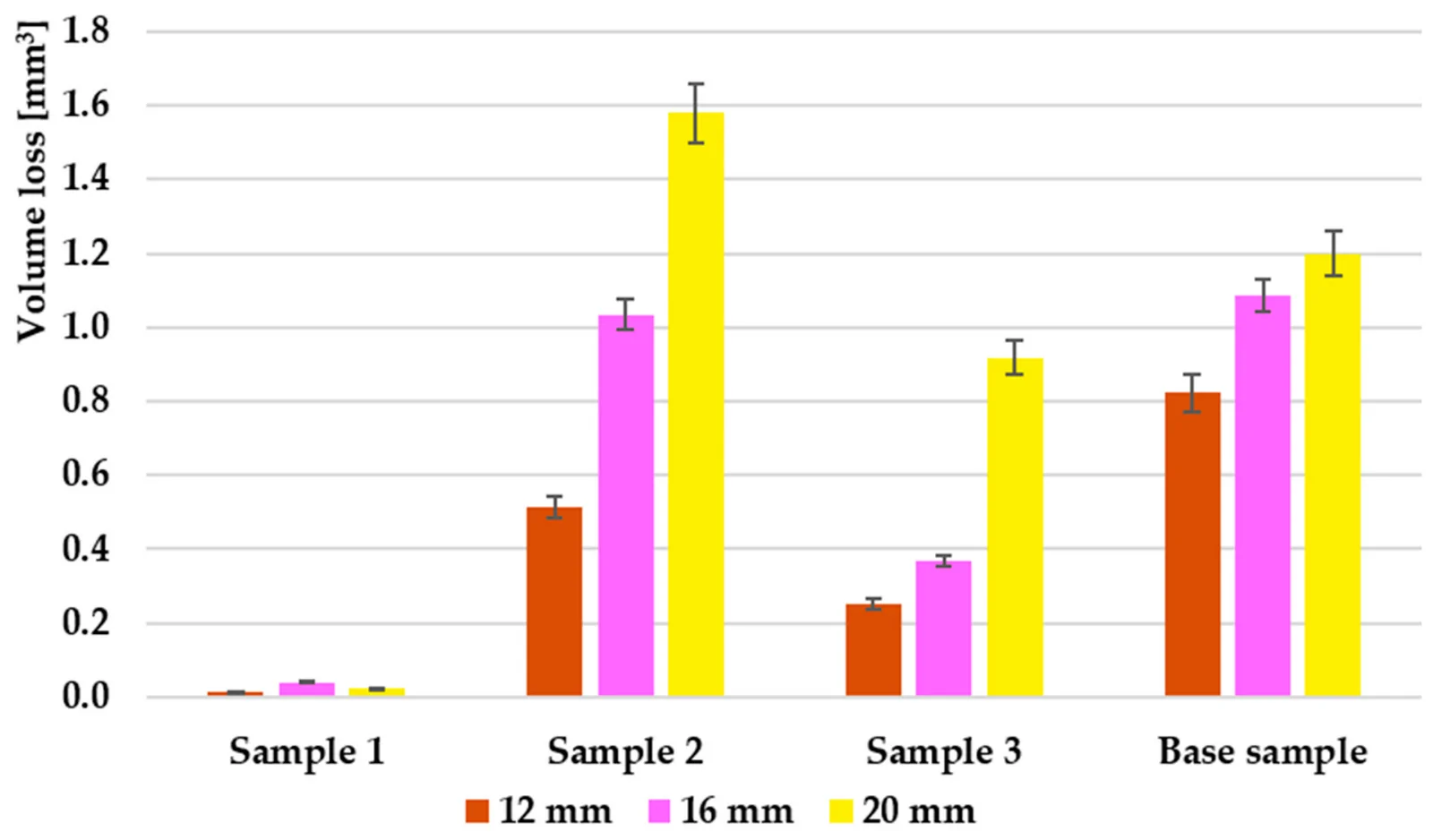
Comparison of volumetric wear at a load of 7.5 N.

**Figure 19:**
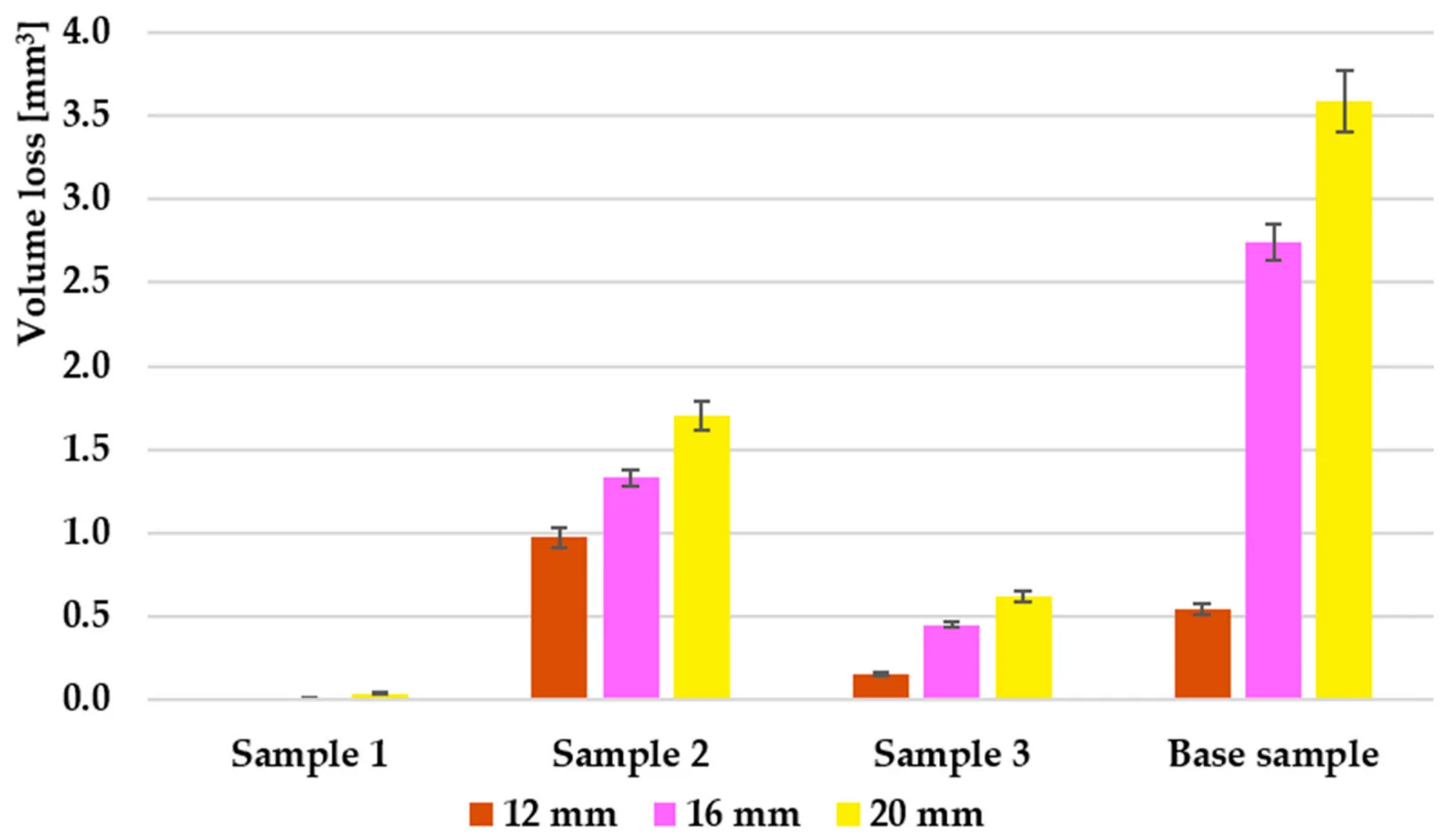
Comparison of volumetric wear at a load of 10 N.

The comparison of volumetric wear loss at loads of 5, 7.5, and 10 N is presented in Figure 17, Figure 18 and Figure 19. For most samples, wear increased with increasing wear-track radius. Increasing the wear-track radius increased the sliding velocity and total sliding distance, thereby increasing the frictional energy dissipated within the contact. However, because the contact temperature and thermal properties of the proprietary coating systems were not determined, thermal softening and temperature-assisted transfer-film stabilization cannot be confirmed. Consequently, frictional heating is considered only a possible secondary factor and was not used as the primary mechanism to explain the observed wear behavior.

Xylan^®^ 1425 (Sample 1) exhibited an almost negligible volumetric loss under all investigated conditions, generally within the range of approximately 0.00–0.04 mm^3^ (Figure 17, Figure 18 and Figure 19). At the most severe test condition, its volumetric loss was only about 0.03 mm^3^, whereas the uncoated substrate reached approximately 3.60 mm^3^, corresponding to a wear reduction of more than 99%. Sample 1 therefore maintained excellent stability even under conditions associated with the highest sliding velocity and potentially the greatest frictional heating. According to the manufacturer’s recommendations, Xylan^®^ 1425 is intended primarily for high-pressure, low-speed applications and exhibits lower temperature resistance than Xylan^®^ 1052 and Xylan^®^ 1010. Its superior wear performance can therefore be attributed mainly to its high mechanical integrity, strong cohesive stability, and ability to maintain an effective lubricating layer rather than to its thermal resistance alone.

For Xylan^®^ 1052 (Sample 2), the volumetric loss increased markedly with increasing wear-track radius. At 10 N, the wear loss rose from approximately 0.96 mm^3^ to 1.70 mm^3^, representing an increase of about 77% (Figure 19). At 7.5 N and the largest track radius, the volumetric loss reached approximately 1.58 mm^3^, which was about 32% higher than that of the uncoated substrate and considerably higher than the values recorded for the other coatings (Figure 18). Although Xylan^®^ 1052 contains a combined PTFE–MoS_2_ solid-lubricant system and is recommended for elevated contact pressures, its deterioration at higher sliding severity is unlikely to result from exceeding its thermal stability limit. A more plausible explanation is the combined effect of increased sliding velocity, cumulative frictional heating, local disruption of coating continuity, and the formation of loose wear particles acting as third-body abrasives.

Xylan^®^ 1010 (Sample 3) exhibited a different dependence on applied load. At the largest track radius, the volumetric loss decreased from approximately 1.53 mm^3^ at 5 N to 0.91 mm^3^ at 7.5 N and subsequently to 0.61 mm^3^ at 10 N, corresponding to an overall reduction of about 60% (Figure 17, Figure 18 and Figure 19). At 10 N, its volumetric loss was approximately 64% lower than that of Sample 2 and about 83% lower than that of the uncoated substrate. As a PTFE-rich resin-bonded dry-film lubricant, Xylan^®^ 1010 may undergo compaction and plastic accommodation under increased contact pressure. A moderate increase in contact temperature may additionally promote local softening, smoothing of surface asperities, and stabilization of the PTFE transfer film. However, because the temperature was not measured directly, this mechanism should be regarded as a plausible interpretation rather than a confirmed thermal effect.

Overall, Xylan^®^ 1425 (Sample 1) exhibited the highest and most stable wear resistance, despite its lower manufacturer-reported temperature limit compared with Xylan^®^ 1052 and Xylan^®^ 1010. The wear results therefore do not follow a simple ranking based on thermal resistance. Instead, they highlight the dominant roles of coating cohesion, mechanical integrity, plastic accommodation of the polymer matrix, and the ability to form and maintain a stable transfer layer.

**Figure 20:**
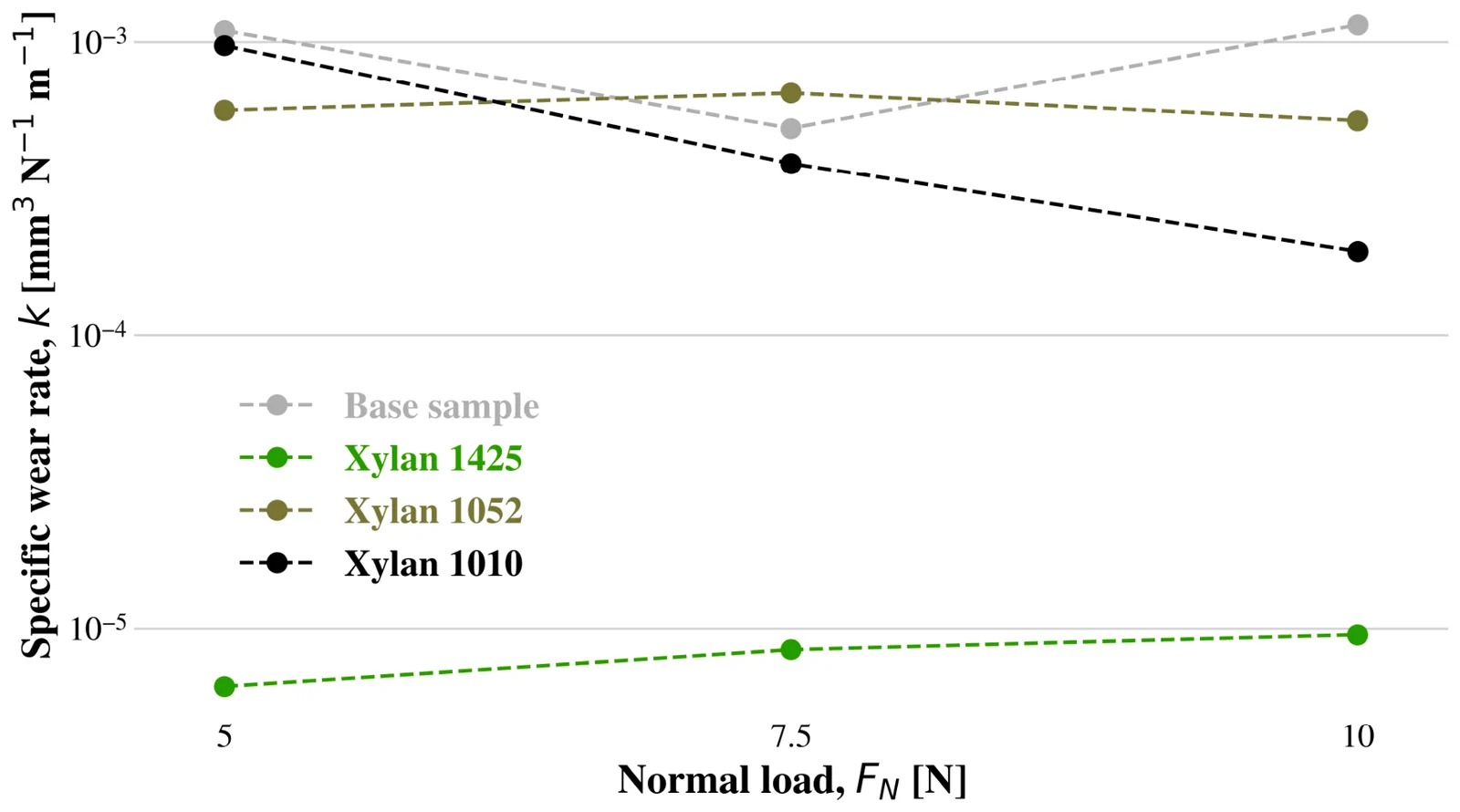
Specific wear rates of the investigated samples at a wear-track radius of 20 mm.

To enable a more objective comparison of wear behavior under different loading conditions, the volumetric wear losses were normalized with respect to the applied normal load and total sliding distance. The specific wear rate was calculated as *k* = V/(F_N_s), where V is the volumetric wear loss, *F_N_* is the normal load, and *s* is the total sliding distance. Figure 20 presents the specific wear rates determined at a wear-track radius of 20 mm. Xylan^®^ 1425 exhibited the lowest values over the entire load range, increasing only slightly from 6.4 × 10^−6^ to 9.5 × 10^−6^ mm^3^·N^−1^·m^−1^. In contrast, Xylan^®^ 1010 showed a decrease from 9.7 × 10^−4^ to 1.9 × 10^−4^ mm^3^·N^−1^·m^−1^ with increasing load. Xylan^®^ 1052 remained within the range of 5.4–6.7 × 10^−4^ mm^3^·N^−1^·m^−1^, while the uncoated base material exhibited comparatively high and non-monotonic values. These normalized results confirm the superior wear resistance of Xylan^®^ 1425.

### 3.5. Wear Mechanism

**Figure 21:**
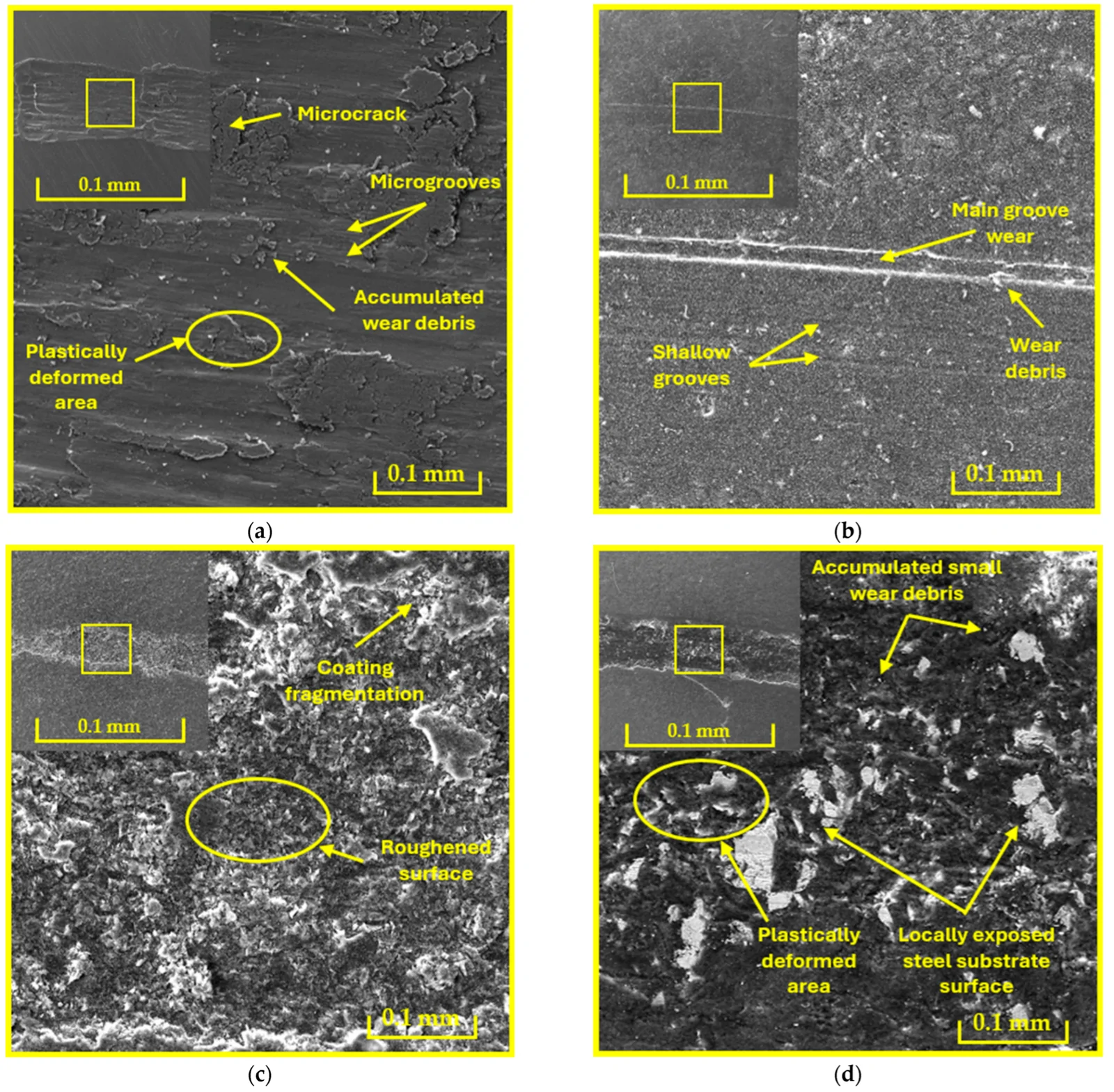
Comparison of the wear mechanisms of the selected specimens tested at a load of 10 N and r = 20 mm: (**a**) uncoated base sample; (**b**) Xylan^®^ 1425 (Sample 1); (**c**) Xylan^®^ 1052 (Sample 2); and (**d**) Xylan^®^ 1010 (Sample 3).

SEM observations of the wear tracks revealed markedly different wear mechanisms for the uncoated steel and the individual PTFE/Xylan coatings, as shown in Figure 21. The uncoated steel surface exhibited pronounced grooves and finer microgrooves oriented predominantly in the sliding direction, together with local microcracking, plastically deformed regions, and accumulated wear debris (Figure 21a). These features indicate a combined adhesive–abrasive wear mechanism. Similar groove formation and debris accumulation are commonly associated with plowing, adhesive junction formation, and the subsequent action of detached particles as third-body abrasives. Sample 1 coated with Xylan^®^ 1425 showed the most uniform and least damaged surface, characterized by a narrow main wear groove, several shallow secondary grooves, and only a limited amount of isolated wear debris (Figure 21b). The surrounding coating remained largely preserved, indicating good coating cohesion and the formation of a stable protective tribofilm. This observation is consistent with the very low volumetric wear of Xylan^®^ 1425. Rodiouchkina et al. similarly demonstrated that extensive and continuous transfer-layer coverage on a steel counterface is associated with reduced friction and improved protection of the polymer surface [31].

Sample 2 coated with Xylan^®^ 1052 exhibited substantially more severe damage, characterized by pronounced coating fragmentation, a strongly roughened wear-track surface, and extensive accumulation and agglomeration of wear debris (Figure 21c). These features suggest progressive cohesive failure of the coating and the participation of detached fragments in third-body abrasion. According to Amenta et al., the formation of large agglomerated debris in PTFE-based materials indicates an unstable and poorly adherent tribolayer, which can contribute to increased friction and progressive material removal [32].

Sample 3 coated with Xylan^®^ 1010 showed pronounced plastic flow, local spalling and delamination, and several bright metallic areas within the wear track (Figure 21d). Based on the additional cross-sectional observations, these metallic areas are interpreted predominantly as locally exposed surfaces of the 18CrNiMo7-6 steel substrate resulting from complete removal of the locally thinned coating. A minor contribution from adhesive transfer of material from the G40 steel ball cannot be entirely excluded; however, it is not considered the primary origin of these areas. The observed morphology therefore indicates a combination of viscoelastic–plastic deformation, fatigue-related damage, localized loss of coating cohesion, and substrate exposure. Overall, the SEM micrographs presented in Figure 21 confirm that Xylan^®^ 1425 exhibited the most stable wear behavior, whereas Xylan^®^ 1052 and Xylan^®^ 1010 showed more pronounced coating disruption, wear-particle formation, and local exposure of the steel substrate.

**Figure 22:**
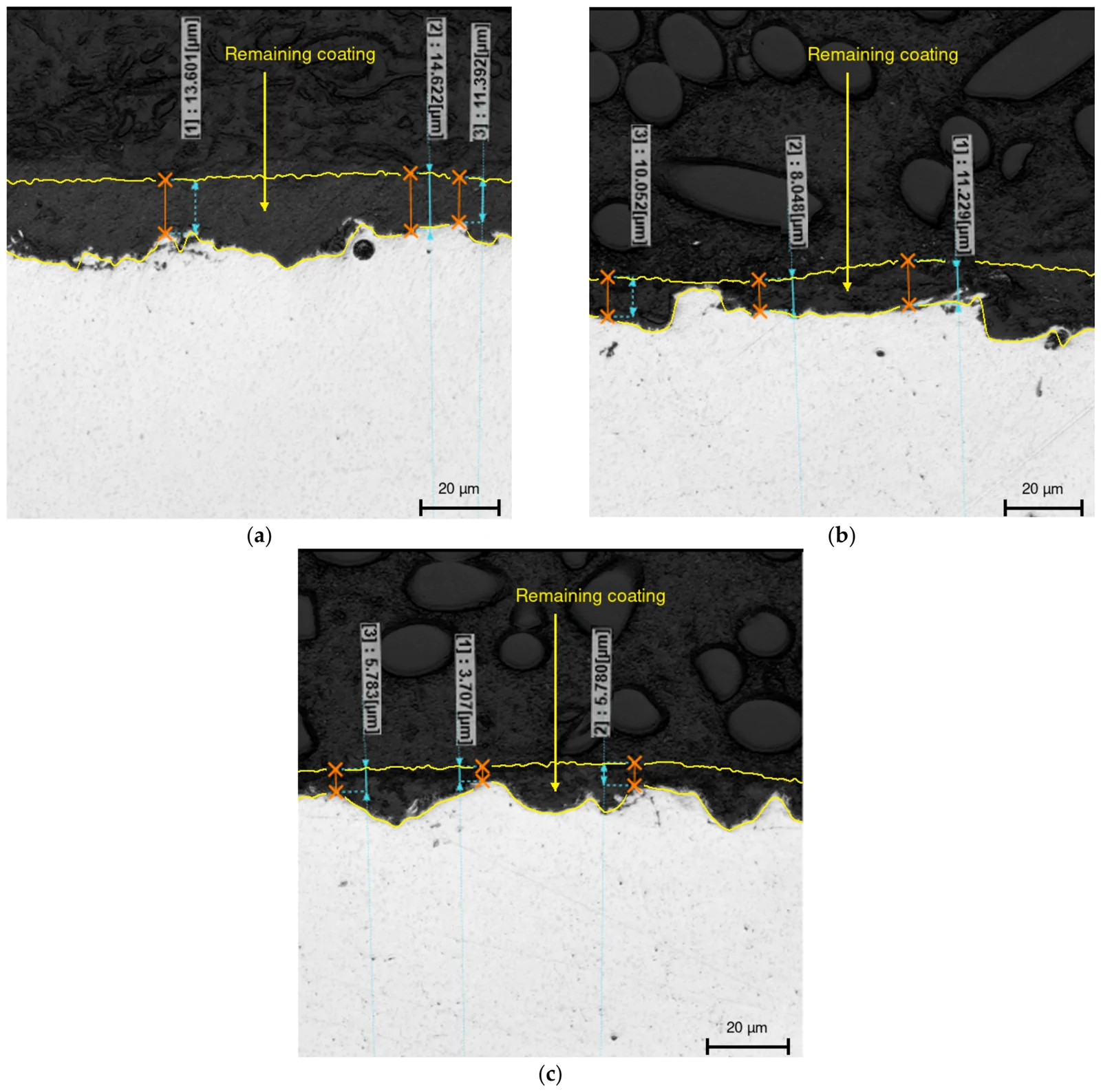
Comparison of cross-sectional micrographs of the wear tracks showing the remaining coating layer and local thickness measurements: (**a**) Sample 1—Xylan^®^ 1425; (**b**) Sample 2—Xylan^®^ 1052; and (**c**) Sample 3—Xylan^®^ 1010.

To clarify the origin of the metallic regions observed within the wear tracks, cross-sectional examinations of the coatings were performed (Figure 22). Sample 1 retained a continuous Xylan^®^ 1425 layer with a thickness of approximately 11.4–14.6 µm (Figure 22a). Samples 2 and 3 exhibited more pronounced local thinning and discontinuities. Despite residual thicknesses of 8.0–11.2 µm for Xylan^®^ 1052 (Figure 22b) and 3.7–5.8 µm for Xylan^®^ 1010 (Figure 22c), the coatings were completely removed at some locations, exposing metallic asperities of the steel substrate. The cross-sections therefore confirm that the metallic regions and Fe signals within the wear tracks originate predominantly from local exposure of the steel substrate rather than exclusively from material transferred from the G40 counterbody. Local residual-thickness measurements do not exclude substrate exposure at adjacent locations because of variations in coating thickness and surface topography.

**Figure 23:**
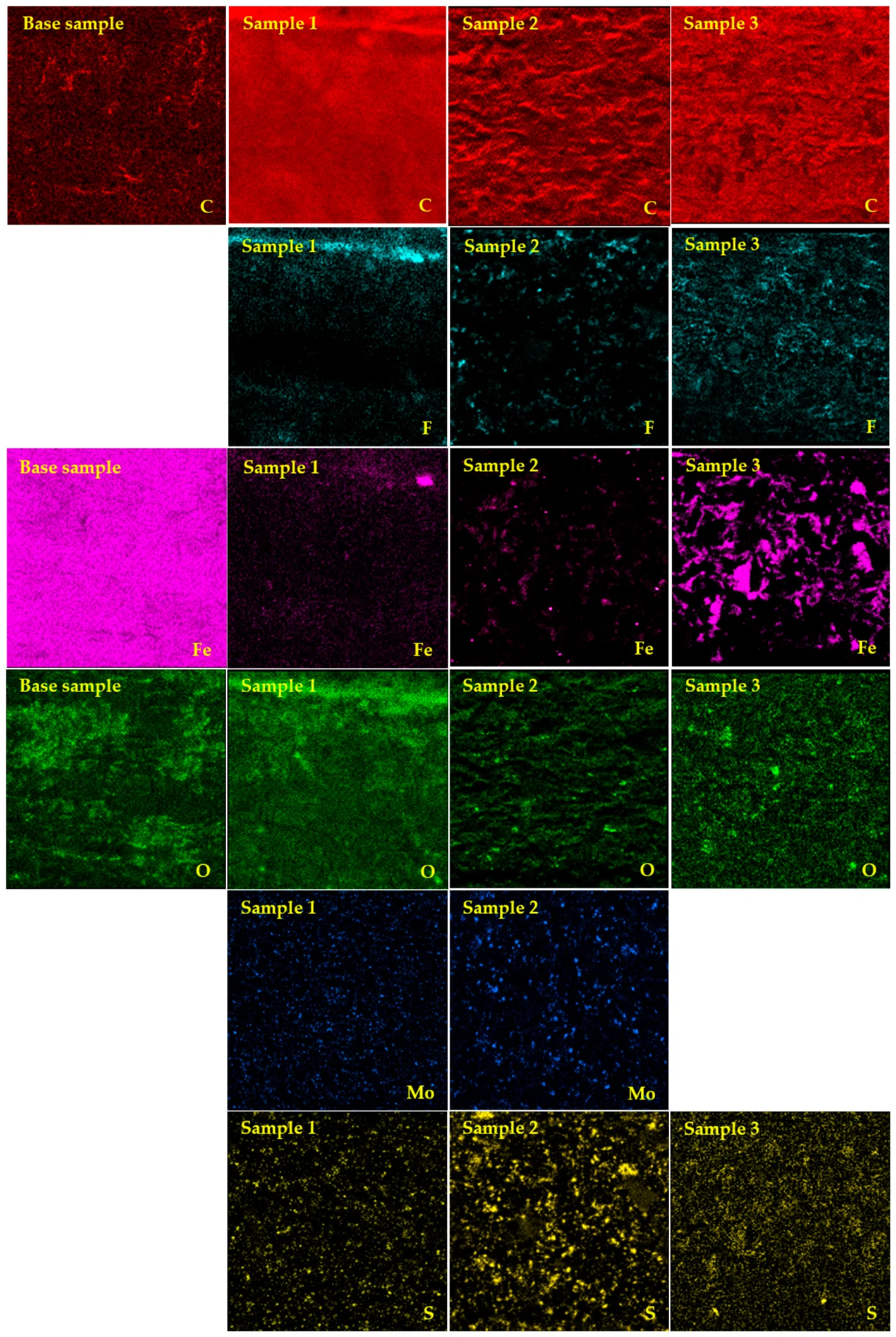
EDS maps of the elemental distribution of selected elements in the wear areas of experimental samples.

EDS mapping was performed in the same wear-track regions as those examined by SEM in Figure 21, while the corresponding combined elemental distribution maps are presented in Figure 23.

For the uncoated base material, a relatively uniform Fe signal predominated, consistent with the chemical nature of the steel substrate. The dispersed C and O signals may be associated with surface contamination, oxidized wear products, and fine debris generated during sliding. Bartošova et al. reported that tribochemical interactions in polymer–metal contacts can promote the formation of iron–oxygen compounds and influence the transition between severe and mild wear regimes [33]. The absence of pronounced F and S signals confirms that these elements are not characteristic constituents of the base material.

For Xylan^®^ 1425 (Sample 1), a continuous distribution of F and C was observed, indicating that the PTFE-based matrix remained largely preserved within the analyzed region. Zeng et al. similarly associated a relatively uniform distribution of C and F in a wear track with the formation and retention of a protective PTFE-rich transfer film [34]. The Fe signal was substantially weaker than that detected for the uncoated substrate. Since the wear-track depth did not exceed the original coating thickness, the detected Fe most likely originated predominantly from adhesive material transfer from the steel counterbody ball rather than from extensive exposure of the substrate. The relatively uniform distributions of Mo and S indicate the preservation of lamellar MoS_2_-containing regions, which may reduce interfacial shear resistance and contribute to transfer-layer stabilization. Tonge et al. likewise demonstrated that Mo- and S-rich tribofilms with a uniform distribution can promote lower friction and improved wear resistance [35]. These observations are consistent with the minimal volumetric wear measured for Sample 1.

Xylan^®^ 1052 (Sample 2) exhibited a more heterogeneous distribution of F, Mo, and S, with these elements concentrated in irregular and locally interrupted regions (Figure 23). Such a distribution indicates local coating fragmentation and redistribution of the PTFE and MoS_2_ phases during sliding. The Fe signal was confined to smaller localized areas. In combination with the cross-sectional evidence of coating thinning, discontinuities, and locally exposed substrate, these Fe-rich areas are attributed primarily to exposure of the 18CrNiMo7-6 steel substrate. A minor contribution from material transferred from the steel ball cannot be completely excluded. This elemental heterogeneity corresponds well with the higher volumetric loss and more pronounced mechanical damage observed for Sample 2. Similar studies of MoS_2_-based solid-film lubricants have shown that discontinuous elemental distributions and localized Fe signals may indicate partial coating removal, non-uniform tribofilm development, and increased wear [35].

For Xylan^®^ 1010 (Sample 3), the distributions of C and F were retained over most of the analyzed area, confirming the presence of a PTFE-rich matrix. No Mo signal was detected, which is consistent with the different composition of this coating. Fe formed locally larger continuous regions corresponding predominantly to locally exposed surfaces of the 18CrNiMo7-6 steel substrate. This interpretation is supported by the cross-sectional observations, which revealed residual coating thicknesses of approximately 3.7–5.8 µm at the measured positions and complete coating removal at adjacent substrate asperities. A wear-track depth lower than the original average coating thickness therefore does not exclude localized substrate exposure because of variations in coating thickness and surface topography. A minor contribution from adhesive transfer of material from the G40 ball cannot be completely excluded but is considered secondary. This interpretation is consistent with the plastic deformation, local coating loss, and substrate exposure observed in the corresponding SEM micrographs.

Overall, the EDS maps and cross-sectional observations confirmed the highest coating continuity for Sample 1, more pronounced fragmentation and localized substrate exposure for Sample 2, and substantial plastic deformation, severe local thinning, and substrate exposure for Sample 3.

## 4. Discussion

The results demonstrate that the tribological performance of the investigated PTFE/Xylan coatings was governed by the combined effects of coating thickness, surface topography, nanomechanical response, binder cohesion, solid-lubricant distribution, and transfer-film stability. Although all coatings reduced the coefficient of friction by approximately 78–80% relative to the uncoated 18CrNiMo7-6 steel, their wear resistance differed substantially. Xylan^®^ 1425 (Sample 1) exhibited the lowest material loss, Xylan^®^ 1010 (Sample 3) produced the lowest COF, and Xylan^®^ 1052 (Sample 2) showed the least favorable wear response among the coated specimens. These findings confirm that low friction and high wear resistance are not necessarily directly correlated.

Coating thickness alone was not decisive. Sample 3 exhibited the greatest initial average thickness, 36.350 ± 1.812 µm, whereas Samples 1 and 2 reached 26.899 ± 1.345 and 26.422 ± 1.321 µm, respectively. Nevertheless, the post-test cross-sectional observations showed that the greater initial thickness of Sample 3 did not prevent severe local thinning and complete coating removal at individual substrate asperities. Residual coating thicknesses of approximately 3.7–5.8 µm were measured at selected positions, whereas the steel substrate was locally exposed at adjacent locations. In contrast, Sample 1 retained a continuous residual coating layer and showed practically negligible volumetric loss under all investigated conditions. Wang et al. similarly reported that the performance of PTFE-based systems depends primarily on the durability, adhesion, and load-bearing capacity of the generated transfer film rather than solely on the amount or thickness of polymeric material [36]. The superior wear resistance of Sample 1 was closely related to its nanomechanical properties. Xylan^®^ 1425 exhibited the highest nanohardness and reduced elastic modulus, reaching 57.02 MPa and 3.33 GPa, respectively, together with the highest values of H/Er = 0.0171 and H3/Er2 = 16.72 × 10^−6^ GPa. These values indicate the greatest resistance to localized permanent deformation and correspond well with the narrow and shallow wear track, whose cross-sectional area was only approximately 556 µm^2^. The smooth transition between the worn and unaffected surfaces and the absence of pronounced edge pile-up further indicate limited plastic flow and high coating cohesion. Conte et al. likewise concluded that favorable PTFE-composite performance requires a balance between a low-shear polymer phase and mechanically stable constituents capable of supporting the applied load [37]. The SEM and EDS results further confirmed this relationship. Sample 1 retained a continuous C- and F-rich matrix and a relatively homogeneous distribution of Mo and S, indicating that the PTFE/MoS_2_-containing phase remained integrated within the coating. Thus, its high wear resistance resulted from the combined action of a mechanically stable binder and a low-shear lubricating phase. Amenta et al. similarly showed that wear resistance depends strongly on whether the transferred material forms a coherent protective tribolayer or coarse, weakly adherent debris [38]. Xylan^®^ 1010 exhibited the lowest nanohardness and elastoplastic indices and therefore the greatest tendency toward viscoelastic–plastic deformation. This was reflected in pronounced pile-up, local spalling, redistribution of material, severe local coating thinning, and exposure of the steel substrate within the wear track. The bright metallic and Fe-rich regions observed on the worn surface are therefore interpreted predominantly as locally exposed areas of the 18CrNiMo7-6 substrate rather than as metallic debris transferred from the G40 steel ball. A minor contribution from counterbody transfer cannot be completely excluded but is considered secondary. The apparently contradictory behavior of Xylan^®^ 1010 demonstrates that a low coefficient of friction does not necessarily imply high resistance to deformation or wear. Its higher initial roughness may have reduced the number of load-bearing microcontacts during running-in, while the PTFE-rich interface and transfer-film formation maintained low interfacial shear resistance. At the same time, its low nanohardness and reduced elastic modulus promoted asperity flattening, plastic flow, pile-up, local spalling and coating removal. Therefore, friction was governed mainly by the low-shear interfacial layer, whereas deformation and wear were controlled by the mechanical integrity and compliance of the coating; initial roughness represented only a secondary factor. This interpretation is consistent with Amenta et al., who demonstrated that friction and wear can follow different trends in PTFE composites [38]. The volumetric loss of Sample 3 at the largest track radius decreased from approximately 1.53 mm^3^ at 5 N to 0.61 mm^3^ at 10 N. This non-linear behavior may be associated with pressure-assisted compaction of the PTFE-rich matrix, smoothing of surface asperities, and stabilization of the transfer film. A contribution from friction-induced softening is also possible, although it cannot be confirmed because the contact temperature was not measured [39]. Wang et al. similarly demonstrated that the wear response of filled PTFE systems depends on the interaction between plastic deformation, filler–matrix bonding, and the stability of transferred material [40].

In contrast, Xylan^®^ 1052 showed pronounced fragmentation despite containing both PTFE and MoS_2_. Its volumetric loss at 10 N increased from approximately 0.96 mm^3^ at the smallest radius to 1.70 mm^3^ at the largest radius. The broad wear track, marked edge pile-up, loose debris, and heterogeneous F, Mo, and S distributions indicate local cohesive failure, non-uniform redistribution of the lubricating phases, and the development of third-body abrasion. The post-test cross-sections additionally revealed local coating thinning, discontinuities, and exposure of the steel substrate, despite residual coating thicknesses of approximately 8.0–11.2 µm at the selected measurement positions. The localized Fe-rich regions observed for Sample 2 are therefore attributed primarily to substrate exposure, although a minor contribution from material transferred from the G40 ball cannot be completely excluded. Qi et al. reported that discontinuity and local detachment of PTFE transfer films significantly increase wear intensity and destabilize tribological behavior [41]. Although Xylan^®^ 1425 and Xylan^®^ 1052 nominally contain both PTFE and MoS_2_, the EDS maps revealed a clear difference in the spatial distribution and retention of the corresponding elements. Xylan^®^ 1425 exhibited a continuous C- and F-rich matrix together with a comparatively fine and homogeneous distribution of Mo and S. In contrast, Xylan^®^ 1052 showed locally interrupted F-rich regions and pronounced clustering of Mo and S, indicating fragmentation and non-uniform redistribution of the solid-lubricant phases during sliding. These observations do not demonstrate a difference in the absolute PTFE or MoS_2_ concentrations or directly identify the binder chemistry. However, when considered together with the substantially higher nanohardness (57.02 versus 31.70 MPa), reduced elastic modulus (3.33 versus 2.78 GPa), and markedly lower volumetric wear of Xylan^®^ 1425, they support the hypothesis that its superior performance originates from greater binder cohesion and more effective retention of the PTFE/MoS_2_ phases. The binder architecture and degree of cross-linking nevertheless remain inferred characteristics that would require complementary spectroscopic or thermal analysis for direct confirmation.

Because the exact constituent fractions and binder formulations remain proprietary, the observed relationships should be interpreted as correlations between the properties of the complete commercial coating systems rather than as direct causal relationships between individual constituents and tribological performance.

Increasing the wear-track radius generally increased volumetric wear because of the associated increase in sliding velocity, total sliding distance, and frictional energy dissipation. This effect was most pronounced for Sample 2, whose mechanically less stable layer progressively fragmented under more severe sliding conditions. Ficici et al. likewise concluded that load and sliding speed can affect friction and wear through different non-linear mechanisms, while composition and transfer-film development remain the principal factors controlling PTFE-composite performance [42].

The additional cross-sectional observations clarify the origin of the Fe-rich regions detected on the coated specimens. Although the measured wear-track depths and residual coating thicknesses at selected positions remained below the original average coating thicknesses, they do not exclude complete coating removal at adjacent locations. Both the initial coating thickness and the wear-track topography were spatially non-uniform. Consequently, the localized Fe-rich and bright metallic regions observed for Samples 2 and 3 are interpreted predominantly as exposed surfaces of the 18CrNiMo7-6 steel substrate. A minor contribution from adhesive transfer from the G40 steel ball and its subsequent incorporation into the polymer matrix remains possible but is considered secondary. The combined SEM, EDS, and cross-sectional results therefore indicate local coating degradation and substrate exposure rather than predominantly dynamic transfer of metallic material from the counterbody.

Independently of the origin of the localized Fe-rich regions, recent studies support the broader importance of PTFE transfer-film formation. Lin et al. demonstrated that the friction-induced formation of a PTFE transfer coating can produce a low-shear interface and markedly reduce friction and wear [43]. Duan et al. showed that the long-term tribological performance of self-lubricating polymer systems also depends on their ability to retain the lubricating phase and maintain a stable boundary film [44]. Although these systems differ from the investigated Xylan^®^ coatings, both studies emphasize the importance of lubricant retention and transfer-film stability in achieving low friction and wear.

Overall, the results show that efficient tribological protection requires two complementary functions: the formation of a low-shear PTFE-rich interface and sufficient structural integrity to retain this lubricating phase under repeated contact loading. Xylan^®^ 1010 provided the lowest friction but underwent pronounced plastic deformation, severe local thinning, and substrate exposure, whereas Xylan^®^ 1052 reduced friction without adequately suppressing coating fragmentation, material loss**, and local substrate exposure**. Xylan^®^ 1425 achieved the most favorable balance between mechanical stability, coating continuity, tribofilm stability, and low interfacial shear, resulting in the highest overall tribological resistance.

## 5. Conclusions

This study evaluated the tribological properties of Xylan^®^ 1425, Xylan^®^ 1052, and Xylan^®^ 1010 coatings applied to 18CrNiMo7-6 steel under dry sliding conditions using the Ball-on-Disk method. The tribological tests were complemented by coating-thickness and surface-roughness measurements, nanoindentation testing, wear-track profilometry, SEM/EDS analysis, and post-test cross-sectional microscopy. Based on the obtained results, the following main conclusions can be drawn:

1. All investigated PTFE/Xylan coatings significantly reduced the coefficient of friction compared with the uncoated steel. The base material exhibited COF values of approximately 0.49–0.64, whereas the coated samples showed values in the range of approximately 0.09–0.13. The average COF reduction reached 79.0% for Xylan^®^ 1425, 78.3% for Xylan^®^ 1052, and 80.3% for Xylan^®^ 1010.
2. Xylan^®^ 1425 exhibited the most favorable nanomechanical response. It achieved the highest nanohardness of 57.02 MPa, reduced elastic modulus of 3.33 GPa, H/Er ratio of 0.0171, and H^3^/Er^2^ value of 16.72 × 10^−6^ GPa. These results confirm its highest resistance to localized and permanent plastic deformation.
3. Xylan^®^ 1425 also exhibited the highest wear resistance. At a load of 10 N and a wear-track radius of 20 mm, the cross-sectional area of the wear track was approximately 556 µm^2^, corresponding to a reduction of about 98% compared with the uncoated steel. Under the most severe test conditions, its volumetric loss was only approximately 0.03 mm^3^, representing a wear reduction of more than 99%. Post-test cross-sectional microscopy additionally confirmed that Xylan^®^ 1425 retained a continuous residual coating layer with a thickness of approximately 11.4–14.6 µm.
4. Xylan^®^ 1010 achieved the lowest coefficient of friction but also exhibited the lowest nanohardness and the greatest susceptibility to viscoelastic–plastic deformation. Pronounced pile-up, local spalling, severe coating thinning, and local exposure of the steel substrate were observed within its wear track. Nevertheless, at the largest wear-track radius, its volumetric wear decreased with increasing load from approximately 1.53 mm^3^ at 5 N to 0.61 mm^3^ at 10 N. This behavior indicates the importance of compaction and redistribution of the PTFE-rich matrix in stabilizing the tribological contact.
5. Xylan^®^ 1052 exhibited the least favorable wear behavior among the coated samples. Its volumetric loss increased markedly with increasing wear-track radius and reached approximately 1.70 mm^3^ at 10 N and r = 20 mm. Profilometric, SEM, and EDS analyses revealed pronounced plastic flow, coating fragmentation, heterogeneous redistribution of F, Mo, and S, and the formation of loose wear debris that may have acted as abrasive third bodies. Cross-sectional observations additionally revealed coating discontinuities, severe local thinning, and localized exposure of the 18CrNiMo7-6 steel substrate.
6. The comparison between Xylan^®^ 1425 and Xylan^®^ 1052 confirmed that coating thickness and the nominal presence of PTFE and MoS_2_ alone are not sufficient indicators of wear resistance. The decisive factors were the mechanical integrity of the binder, coating cohesion, the uniformity of solid-lubricant distribution and retention, and the formation and stability of a protective tribofilm.
7. The combined SEM, EDS, and cross-sectional observations clarified the origin of the Fe-rich metallic regions detected within the wear tracks. For Xylan^®^ 1052 and Xylan^®^ 1010, these regions originated predominantly from local exposure of the 18CrNiMo7-6 steel substrate following severe coating thinning or complete local removal. A minor contribution from material transferred from the G40 steel counterbody cannot be completely excluded but is considered secondary.

Overall, Xylan^®^ 1425 provided the most favorable combination of a low coefficient of friction, high nanomechanical resistance, coating-layer continuity, and minimal material loss. In contrast to Xylan^®^ 1052 and Xylan^®^ 1010, it preserved a continuous residual coating layer without evidence of extensive substrate exposure. The obtained results therefore indicate that, among the investigated coatings, it represents the most suitable solution for protecting 18CrNiMo7-6 steel components operating under dry sliding conditions within the evaluated ranges of normal load and sliding velocity.

## Figures and Tables

**Table 1:** Chemical composition (wt.%) of steel 18CrNiMo7-6.

Element	C	Si	Mn	P	S	Cr	Ni	Mo	Cu	Fe
18CrNiMo7-6/1.6587	0.15–0.21	max. 0.40	0.50–0.90	max. 0.025	max. 0.035	1.50–1.80	1.40–1.70	0.25–0.35	max. 0.40	Balance
Spectral analysis	0.20	0.19	0.85	0.003	0.005	1.70	1.65	0.28	0.14	Balance

**Table 2:** Marking of samples used PTFE/Xylan coatings.

Sample Designation	Coating Designation	Operating Temperature [°C]	Coating Color
Base Sample	Uncoated	–	–
Sample 1	Xylan^®^ 1425	−20 to +180	Green
Sample 2	Xylan^®^ 1052	−195 to +260	Dark green
Sample 3	Xylan^®^ 1010	−195 to +260	Black

**Table 3:** Chemical composition (wt.%) of steel G40 (equivalent to 100Cr6/1.3505).

Element	C	Cr	Mn	Si	P	S	Fe
G40//100Cr6	0.93–1.10	1.30–1.65	0.20–0.45	0.15–0.35	max. 0.030	max. 0.025	Balance
Spectral analysis	1.06	1.52	0.27	0.17	0.003	0.003	Balance

**Table 4:** Experimental parameters of the Ball-on-Disk tribological tests.

Load [N]	Wear Track Radius [mm]	Sliding Speed [m·s^−1^]	Rotational Speed [min^−1^]	Time [s]
5	12	0.31	250	600
	16	0.41		
	20	0.52		
7.5	12	0.31		
	16	0.41		
	20	0.52		
10	12	0.31		
	16	0.41		
	20	0.524		

**Table 5:** Nanoindentation properties and elastoplastic indices of the investigated PTFE/Xylan coatings.

Sample	Nanohardness (H) [MPa]	Reduced Elastic Modulus (E_r_) [GPa]	H/E_r_ [−]	H^3^/E_r_^2^ [GPa]
Sample 1	57.02	3.33	0.0171	16.72 × 10^−6^
Sample 2	31.70	2.78	0.0114	4.12 × 10^−6^
Sample 3	25.27	2.66	0.0095	2.28 × 10^−6^

## Data Availability

The original contributions presented in this study are included in the article. Further inquiries can be directed to the corresponding authors.
